# Itaconate Modulates Neutrophil Homeostasis to Ameliorate Airway Inflammation in Diesel Exhaust Particles-exacerbated Asthma via Inhibiting NETs Formation

**DOI:** 10.7150/ijbs.124927

**Published:** 2026-02-11

**Authors:** Guiping Zhu, Ling Ye, Yansha Song, Yu Chen, Hui Cai, Zilinuer Abuduxukuer, Liping Zhu, Yingying Zeng, Wenjiao Zhu, Dan Ye, Yuanlin Song, Pu Wang, Meiling Jin, Jian Wang

**Affiliations:** 1 Department of Allergy, Zhongshan Hospital, Fudan University, Shanghai 200032, China.; 2 Department of Pulmonary and Critical Care Medicine, Zhongshan Hospital, Fudan University, Shanghai 200032, China.; 3 Molecular and Cell Biology Lab, Institutes of Biomedical Sciences, Shanghai Medical College of Fudan University, Shanghai 200032, China.

**Keywords:** Itaconate, Corticosteroid-resistant asthma, Neutrophils, Neutrophil extracellular traps, Diesel exhaust particles

## Abstract

Particulate matter exposure, especially diesel exhaust particles (DEP), can exacerbate neutrophilic airway inflammation which presents corticosteroid insensitivity, resulting in the loss of asthma control. The underlying biological mechanisms remain poorly understood, thereby impeding the development of innovative therapeutic strategies. Itaconate (ITA) is an anti-inflammatory metabolite that suppresses excessive immune activation in multiple pathological conditions. In this study, we identified that neutrophil acted as an essential regulator in DEP-induced corticosteroid-resistant asthma mouse models. Multi-omics and single-cell sequencing analysis found that aconitate decarboxylase 1 (ACOD1)/ITA was significantly elevated in neutrophils via the NF-κB signaling pathway in DEP-exacerbated asthma. Knockout of *Acod1* exacerbated asthma pathogenesis, while treatment with exogenous ITA or 4-octyl itaconate (4-OI) conferred protection against airway inflammation and reversed corticosteroid resistance in asthma mouse models. Mechanistically, neutrophil-derived ITA helped maintain immune homeostasis by reducing the formation of neutrophil extracellular traps (NETs), which further inhibited Th17 cell differentiation in DEP-exacerbated asthma. Our results delineate the dual immunoregulatory function of neutrophils in DEP-induced corticosteroid-resistant asthma, wherein they simultaneously propagate inflammation through NETosis and Th17 activation while restraining immune hyperactivation via ITA-mediated metabolic regulation. ITA serves as a negative regulator of airway inflammation and corticosteroid resistance, highlighting its promising therapeutic potential in asthma.

## Introduction

Asthma is a heterogeneous chronic airway disease affecting over 300 million individuals globally, pathologically characterized by reversible airflow obstruction 1. Asthma pathogenesis involved a complex interplay of genetic susceptibility, immune dysregulation, and environmental exposures, with particular susceptibility to airborne pollutants 2. Particulate matter (PM), a ubiquitous component of air pollution, constitutes a major environmental risk factor for asthma exacerbations and mortality 3-5. Exposure to diesel exhaust particles (DEP), a predominant component of the traffic-related particulate matter, has been demonstrated to exacerbate asthma severity and impair symptom control in both *in vitro* and *in vivo* studies 6. Corticosteroids serve as the cornerstone of asthma management, with treatment regimens combining inhaled corticosteroids with long-acting β-agonists offering effective control for the majority of patients. However, approximately 5% to 10% of asthma patients exhibit corticosteroid insensitivity in clinical practice, and this subgroup significantly contributes to the overall health burden and mortality associated with asthma 7, 8. The etiology of corticosteroid resistance in asthma remains incompletely understood. However, growing evidence implicates air pollution exposure, particularly DEP, as a significant risk factor of corticosteroid-resistant asthma development 9, 10.

Corticosteroid-resistant asthma is typically classified into two main endotypes: type 2 (T2) and non-type 2 (non-T2) asthma 11-13. While biologics have emerged as promising therapies for T2 corticosteroid-resistant asthma, effective therapeutic interventions for non-T2 asthma remains substantially underdeveloped 7. Neutrophilic inflammation represents a critical subtype of non-T2 asthma, and neutrophil depletion was reported as an approach for reversing corticosteroid resistance 14. The pathogenetic mechanism of neutrophils in corticosteroid-resistant asthma involves promoting Th17 cell differentiation. However, the precise mechanisms governing neutrophil-Th17 cell crosstalk in this context remain incompletely characterized, presenting a critical knowledge gap in understanding corticosteroid-resistant asthma pathogenesis.

Itaconate (ITA) is an endogenous immune-metabolite that serves as a critical regulator in preventing immune overactivation in diverse disease contexts 15. ITA is uniquely produced by myeloid cells, including macrophages and neutrophils 16. The induced expression of aconitate decarboxylase 1 (ACOD1) upon various inflammatory stimuli promotes the production of ITA from the TCA intermediate metabolite cis-aconitate 17. Numerous studies indicate that ITA exerts its anti-inflammatory effect through various mechanisms, including inhibition of succinate dehydrogenase, promoting alkylation of proteins like KEAP1 and JAK1, and competitively inhibiting DNA demethylase TET2 to induce epigenetic reprogramming 18-21. However, the potential of ITA to confer protection in corticosteroid-resistant asthma remains unexplored.

This study seeks to delineate the role of ITA in DEP-exacerbated asthma and to elucidate the underlying biological mechanisms of corticosteroid resistance. We constructed the mouse models of corticosteroid-resistant asthma aggravated by DEP exposure, and further used multi-omics and single-cell sequencing analysis to reveal the metabolic reprogramming in corticosteroid-resistant asthma. Neutrophil-derived ITA was identified to act as a critical regulator of immune and inflammatory homeostasis and highlight its potential therapeutic value in the management of corticosteroid-resistant asthma.

## Materials and Methods

### Mice

Female C57BL/6J mice (6-8 weeks old) were purchased from Vital River Laboratory Animal Technology Co. (Beijing, China) and housed under specific pathogen-free (SPF) conditions at the Animal Center of Zhongshan Hospital, Fudan University. The *Acod1^-/-^* mice were generously provided by Professor Ye Dan from Shanghai Medical College of Fudan University.

### Human samples

Blood samples or bronchoalveolar lavage fluid (BALF) samples were obtained from healthy donors, mild-to-moderate asthma patients, and severe asthma patients in accordance with the protocol approved by the Ethics Committee of Zhongshan Hospital, Fudan University (B2019-020R). Peripheral blood mononuclear cells (PBMCs) were isolated from whole blood samples by Ficoll-Paque density gradient centrifugation and then cryopreserved. BALF supernatant was collected, and cell smears were prepared using cytospin slides.

### Establishment of corticosteroid-resistant asthma mouse model and intervention studies

To develop a mouse model of corticosteroid-sensitive asthma induced by house dust mite (HDM) and corticosteroid-resistant asthma induced by HDM in conjunction with DEP, mice were sensitized via intratracheal (i.t.) administration of HDM (10 μg in 40 μL PBS; Greer Laboratories) on days 0-4, followed by daily challenge with HDM (10 μg), with or without DEP (100 μg; NIST), in 40 μL PBS from days 10 to 13. The model of corticosteroid-resistant asthma induced by HDM and lipopolysaccharide (LPS) (Sigma, USA) was established by intranasal administration of HDM (20 μg) and LPS (5 μg) during sensitization stage on days 0-2, followed by HDM (10 μg) during the challenge stage on days 9-13. Control mice received PBS following the same protocol.

For intervention studies in the HDM, HDM/DEP, or HDM/LPS models, mice received ITA (pH = 7, 0.25 mg/kg, intranasally; MedChemExpress, USA), or 4-octyl itaconate (4-OI) (50 mg/kg, intraperitoneally; MedChemExpress, USA) 1 hour prior to each challenge. Dexamethasone (DEX) (1 mg/kg; Sigma, USA) was administered intraperitoneally as a positive control in the models. Anti-IL-17A monoclonal antibody (BioXcell, USA) or the isotype control antibody was administered intraperitoneally at the dose of 100 μg/mouse on days 10-13, 1 hour prior to each challenge. For neutrophil depletion, 0.5 mg of anti-Ly6G antibody (BioXcell, USA) or the isotype control antibody was administered intraperitoneally on days 10-13, also 1 hour before each challenge. Additionally, DNase I treatment (1 mg/kg; MedChemExpress, USA) was administered intraperitoneally one hour prior to each challenge.

All mice were euthanized for sample collection on day 14. All animal procedures were conducted following institutional guidelines and approved by the Animal Care and Use Committee of Zhongshan Hospital, Fudan University (No. 2023-297).

### BALF collection and flow cytometry detection

BALF was collected by instilling 1 mL of PBS into the lungs via a tracheal cannula, followed by gentle aspiration. This procedure was performed in triplicate to maximize recovery. The recovered fluid was subsequently centrifuged, with the supernatant stored at -80 °C for later analysis.

The cell pellets were resuspended in PBS and subjected to flow cytometric analysis utilizing the following fluorochrome-conjugated antibodies: Zombie Aqua™ dye, CD45, Ly6G, CD11c, Siglec-F, and CD11b (all from BioLegend, USA) (Figure S1A). Total cell counts in BALF were determined using the CellDrop® cell counter (DeNovix, USA). Aria Ⅲ flow cytometer (BD Biosciences, USA) was used to analyze the stained cells, and the data were analyzed with FlowJo version 10 software.

### Histological staining

Lung tissues were fixed in 4% paraformaldehyde. Tissue sections were stained with hematoxylin and eosin (H&E) and periodic acid-Schiff (PAS) after being embedded in paraffin.

### Flow cytometry of Th2 and Th17 in the lung tissues

Lung tissues were digested with 1 mg/mL Collagenase IV and 20 μg/mL DNase I at 37 °C for 30 min. Following filtration and treatment with red blood cell lysis solution, single-cell suspensions were stimulated for five hours using a leukocyte activation cocktail (BD Pharmingen, USA). The cells were then stained with anti-CD16/32 (BioLegend, USA) for 15 min, followed by staining with Zombie Aqua™ dye (BioLegend, USA) at room temperature for 15 min. Cells were stained with a mixture of CD45 (BioLegend, USA), CD3e (BioLegend, USA), and CD4 (BioLegend, USA) for 30 min on ice. For detection of Th2 and Th17 intracellular cytokines, cells were treated with the Perm/Wash buffer kit (BD Pharmingen, USA) according to the manufacturer's instructions and stained with fluorochrome-conjugated antibodies against IL-4 (Biolegend, USA) or IL-17A (Biolegend, USA). Aria Ⅲ flow cytometer (BD Biosciences, USA) was used to analyze the stained cells, and the data were analyzed with FlowJo version 10 software (Figure S1B).

### Cytokine measurement

Lung tissues were homogenized in Western and IP lysis buffer (Beyotime, China) containing protease inhibitors, and protein concentrations were assessed using a NanoDrop Microvolume spectrophotometer (Thermo Fisher, Germany). The levels of IL-4, IL-6, and IL-17A in the BALF and lung tissues were quantified with a BD™ CBA Mouse Th1/Th2/Th17 Cytokine Kit, in accordance with the manufacturer's protocol. Briefly, 50 μL of sample or cytokine standards were incubated with capture beads and PE-conjugated detection antibodies at room temperature for 2 hours in the dark. Samples were washed and analyzed using Aria III flow cytometer (BD Biosciences, USA), and data were processed using FCAP Array™ software. In some experiments, BALF were measured by ELISA following the manufacturer's instructions (MultiSciences, China).

### Metabolomics analysis

Frozen lung tissue samples (≥ 50 mg), stored at -80 °C, were homogenized in 1 mL of pre-chilled methanol: water (4:1, v/v) using a cryogenic grinder operating at 60 Hz for 3 min. Homogenates were incubated at -80 °C for 12 h. After centrifugation at 14,000 rpm for 10 min at 4 °C, the supernatants were collected and vacuum-dried. The dried extracts were then resuspended in pre-chilled 50% methanol and subjected to centrifugation at 14,000 rpm for 10 min. The resulting supernatant underwent liquid chromatography-tandem mass spectrometry (LC-MS/MS) analysis using a Vanquish UHPLC system (Thermo Fisher, Germany) coupled with an Orbitrap Q Exactive™ HF-X mass spectrometer (Thermo Fisher, Germany). Data acquisition was performed in both positive and negative ion scanning modes. The identification of metabolites was carried out through the BGI reference library (Shenzhen, China), the KEGG database, HMDB database, and Metlin database. Principal component analysis (PCA) and orthogonal partial least squares discrimination analysis (OPLS-DA) were performed to elucidate differences among various groups and explore underlying metabolic variations. Differentially accumulated metabolites (DAMs) were defined based on variable importance in projection (VIP) values obtained from the OPLS-DA model ≥ 1.0, with a significance threshold of P-value < 0.05 and a fold change criterion of ≥ 1.2 or ≤ 0.83.

### RNA-sequencing

Total RNA of the lung tissues was extracted using the TRIzol Reagent (Thermo Fisher Scientific, USA). The RNA sequencing was conducted by the Beijing Genomics Institute (BGI) using the DNBSEQ platform after rigorous qualitative and quantitative assessments. Differentially expressed genes (DEGs) between groups were analyzed by DESeq2, with a false discovery rate (FDR) < 0.05 and fold change ≥ 2.

### Measurement of ITA by liquid chromatography-mass spectrometry (LC/MS)

Lung tissue samples (≥ 50 mg) were stored at -80 °C before analysis. Samples were homogenized in 1 mL of pre-chilled methanol: water (4:1, v/v) that had been equilibrated at -80 °C for 4-6 hours using a cryogenic grinder set to 60 Hz for 3 min. Homogenates were incubated at -80 °C for 12 h. After centrifuging at 14,000 rpm for 10 min, the supernatants were collected and concentrated under vacuum. A volume of 200 μL of BALF supernatant was mixed with 800 μL of cold methanol. Samples were then incubated at -20 °C for 1 h before being centrifuged at 14,000 rpm for 10 min at 4 °C. The supernatant was transferred to a fresh 1.5 mL tube and subjected to vacuum drying.

Neutrophils were collected from 6-well plates by centrifugation at 1,000 rpm for 5 min, preserving both the cell pellets and the supernatants. Cell pellets were extracted with 1 mL of pre-chilled 80% methanol (-80 °C), while 200 μL of supernatant was mixed with 800 μL of pre-chilled methanol (-80 °C). All samples were incubated at -20 °C for 1 h, followed by centrifugation at 14,000 rpm for 15 min at 4 °C. The supernatants were transferred to fresh 1.5 mL tubes and vacuum-dried.

All samples were resuspended before detection. To quantify ITA concentrations, a standard calibration curve was created and analyzed alongside the samples utilizing an ultra-high-performance liquid chromatograph (AcquityUPLC I-Class, Waters) coupled with a triple quadrupole mass spectrometer (XevoTQ-XS, Waters).

### Single-cell RNA sequencing (scRNA-seq) and analysis

Single-cell suspensions were prepared from lung tissues, with viability exceeding 90% as determined by trypan blue staining. The sequencing and bioinformatics analysis were provided by OE Biotech Co., Ltd. (Shanghai, China). Briefly, single-cell cDNA libraries were constructed using the MobiNova single-cell sequencing platform. Subsequently, the constructed libraries were sequenced on the Illumina Novaseq 6000 EP150 platform. The raw sequencing data were then aligned to the mouse reference genomes (mouse: GRCm39) using MobiVision (version 3.2) to generate gene expression matrix. After conducting quality control and dimensionality reduction, SingleR was employed for cell type annotation. Cells were visualized using a 2-dimensional Uniform Manifold Approximation and Projection (UMAP) algorithm with the RunUMAP function. Gene visualization and KEGG pathway analysis were executed using Seurat, and cell-to-cell communication was assessed using CellChat.

### Immunofluorescence staining of ACOD1

To evaluate the expression and cellular localization of ACOD1 in the lung tissue, we performed immunofluorescence staining on paraffin-embedded lung sections. The lung sections were incubated with primary antibodies against ACOD1 (Abcam, UK), Ly6G (Servicebio, China), and CD68 (Servicebio, China), followed by incubation with fluorescently-labeled secondary antibody. Nuclear staining was performed with DAPI (Beyotime, China). Ly6G was used to mark neutrophils, and CD68 was used to mark macrophages. For the staining of cell smears of BALF, CD16b was used to mark neutrophils, and CD68 was used to mark macrophages. Immunofluorescence images were subsequently captured using a fluorescence microscope.

### Multi-omics analysis

The conjoint analysis of metabolomics and transcriptomics data was performed using MetaboAnalyst 5.0 by importing DAMs and DEGs into the Joint-Pathway Analysis module. Significantly enriched pathways were determined based on a P-value < 0.05.

### Isolation of neutrophils and purity detection

Mouse bone marrow-derived neutrophils (BMDNs) were isolated using the EasySep™ Mouse Neutrophil Enrichment Kit (Stemcell Technologies, Canada), following the manufacturer's instructions. Peripheral blood neutrophils (PBNs) were isolated using the Human Neutrophil Isolation Kit (Solarbio, China), adhering to the recommended protocol.

BMDNs or PBNs were collected and resuspended in PBS containing 2% fetal bovine serum (FBS; Gibco, USA). Cells were then incubated with CD45, CD11b, and Ly6G (all from BioLegend, USA) for BMDNs, and with CD16, CD66b (both from BioLegend, USA) for PBNs on ice for 30 min. BMDNs were identified as CD45^+^CD11b^+^Ly6G^+^ cells for purity analysis. PBNs were identified as CD16^+^CD66b^+^.

### Culture and treatment of neutrophils

BMDNs harvested from WT or *Acod1^-/-^* mice were cultured in RPMI 1640 supplemented with 10% FBS (Gibco, USA) and 50 U/mL penicillin and streptomycin (Gibco, USA) at 37 °C in cell culture incubator containing 5% CO_2_. BMDNs were treated with either DEP (100 μg/mL) or LPS (100 ng/mL) for specified time intervals. In some experiments, inhibitors targeting toll-like receptor 2 (TLR2) (C29, 100 µM; MedChemExpress, USA), TLR4 (TAK242, 100 nM; MedChemExpress, USA), the NF-κB signaling pathway (Bortezomib, 50 nM; MedChemExpress, USA), as well as ITA or 4-OI, were administered one hour prior to DEP or LPS treatment.

### RNA isolation and real-time qPCR

Total RNA was extracted using the RNA-Quick Purification Kit (ES Science, China) following the manufacturer's protocol. The concentration and purity of the RNA were evaluated by a NanoDrop Microvolume spectrophotometer (Thermo Fisher, Germany). The cDNA was synthesized using the PrimeScript™ RT Reagent Kit (Takara, Japan) according to the manufacturer's instructions. Quantitative PCR was subsequently performed on a QuantStudio™ 5 Real-Time PCR System (Thermo Fisher, Germany) using Hieff® qPCR SYBR Green Master Mix (Yeasen, China). The primer sequences were provided in Table S1.

### Collection of neutrophil extracellular traps (NETs)

Neutrophils were stimulated with DEP (100 μg/mL) for 12 h in 12-well plates, with or without pre-treatment with ITA, or stimulated with phorbol myristate acetate (PMA, 100 nM; MedChemExpress, USA) for 4 h in a 100 mm flat tissue culture dish. After incubation, the supernatant was carefully aspirated following two washes with cold PBS. The collected PBS were centrifuged at 500 g for 10 min at 4 °C to remove cells and debris to obtain cell-free NETs-rich supernatant. For high-concentration of NETs, the supernatant was subjected to a second centrifugation at 18,000 g for 20 min at 4 °C. The pellets were resuspended in PBS and stored at -80 °C until further use.

### Measurement of double-stranded DNA (dsDNA)

The concentration of dsDNA was quantified using the Quant-iT™ PicoGreen™ dsDNA Assay Kit (Thermo Fisher, Germany) according to the manufacturer's instructions. Fluorescence was measured with a microplate reader (excitation/emission: 480/520 nm), and dsDNA concentrations were determined based on a standard curve.

### Immunofluorescence detection of NETs

Neutrophils were cultured on poly-L-lysine-coated coverslips in a 6-well plate and subsequently stimulated with DEP (100 μg/mL) for 12 hours. ITA was added 1 h before DEP stimulation. Cells were fixed with 4% paraformaldehyde for 20 min following stimulation. After fixation, the cells were washed with PBS and permeabilized with 0.5% Triton X-100. The cells were then incubated overnight at 4 °C with primary antibodies against citrullinated histone H3 (CitH3) (Abcam, UK) and MPO (Servicebio, China). The slides were incubated for 1 h with TYR-520 or TYR-570-labeled secondary antibodies. Nuclei were stained with DAPI, and fluorescence microscopy was employed for imaging.

For lung tissue analysis, paraffin-embedded lung sections were incubated with primary antibodies against CitH3 and MPO, followed by the incubation of TYR-520 or TYR-570-labeled secondary antibodies. Nuclei were stained with DAPI, and the slides were imaged using fluorescence microscopy.

### Immunohistochemistry Staining

Lung tissues were fixed in 4% paraformaldehyde and then embedded in paraffin. Immunohistochemical analysis was performed using a rabbit anti-peptidyl arginine deiminase 4 (PAD4) antibody (Proteintech, USA) to evaluate the expression levels of PAD4.

### Western blot

Lung tissue or cell lysates were prepared using RIPA buffer (Beyotime, China) containing phosphatase inhibitors (Roche, Germany) and Phenylmethylsulfonyl fluoride (PMSF; Beyotime, China). Proteins were then separated by sodium dodecyl sulfate-polyacrylamide gel electrophoresis (SDS-PAGE) and transferred to the polyvinylidene difluoride (PVDF) membrane (Millipore, USA). After blocking, the membranes were incubated with primary antibodies against CitH3 (Abcam, UK), PAD4 (Proteintech, USA), TLR2 (ABclonal, China), TLR4 (ABclonal, China), NF-κB (CST,USA), p-NF-κB (CST,USA), ACOD1 (Abcam,UK) or β-actin (Beyotime, China) at 4 °C overnight, followed by incubation with a secondary antibody (Goat anti-mouse or anti-rabbit IgG HRP-conjugated) at room temperature for 1 hour. Target protein detection was performed using ECL detection reagent (Beyotime, China).

### Isolation of naive CD4^+^ T cells

Naive CD4^+^ T cells were isolated from mouse spleen using the Mouse Naive CD4^+^ T Cell Iso Kit (Stemcell Technologies, Canada), in accordance with the manufacturer's instructions. Mouse Naive CD4^+^ T Cells were stained with CD4 (BioLegend), CD44 (BioLegend), and CD62L (BioLegend), gated as CD4^+^CD44^-^CD62L^+^. Human naive CD4^+^ T cells from PBMCs were isolated using the Human naive CD4^+^ T Cell Iso Kit (Stemcell Technologies, Canada) as we previously conducted 22.

### Co-culture of naive CD4^+^ T Cells with neutrophils

Naive CD4^+^ T cells were cultured under Th17 differentiation conditions. Briefly, 96-well U-bottom plates were coated overnight at 4 °C with anti-CD3 (2 μg/mL in PBS, 50 μL/well; BioGems, USA), followed by two washes with PBS. The Th17 differentiation culture medium consisted of anti-CD28 (5 μg/mL; BioGems, USA), TGF-β (5 ng/mL; Peprotech, USA), IL-6 (20 ng/mL; Peprotech, USA), and IL-23 (10 ng/mL; Peprotech, USA). Naive CD4^+^ T cells (2 × 10^5^ cells/well) were either cultured alone or co-cultured with neutrophils at a 1:1 ratio under Th17 differentiation condition. After 6 hours, ITA (6 mM) was added to the appropriate wells. One hour later, cells were stimulated with DEP (100 μg/mL) or NETs (500 ng/mL). After 72-96 hours, cells were harvested for flow cytometry analysis.

### Statistical analysis

Statistical analyses and data visualization were performed using GraphPad Prism (v9.0). Unless otherwise specified, continuous variables are presented as mean ± SEM. Statistical comparisons between two groups were carried out by unpaired Student's t-test. One-way ANOVA followed by Tukey's multiple comparisons test was used for comparing more than two groups. The Pearson correlation test was used for correlation analysis. A P-value < 0.05 was considered statistically significant.

## Results

### DEP exposure promotes neutrophils and Th17 cells-mediated corticosteroid-resistant airway inflammation in asthma

**Figure 1:**
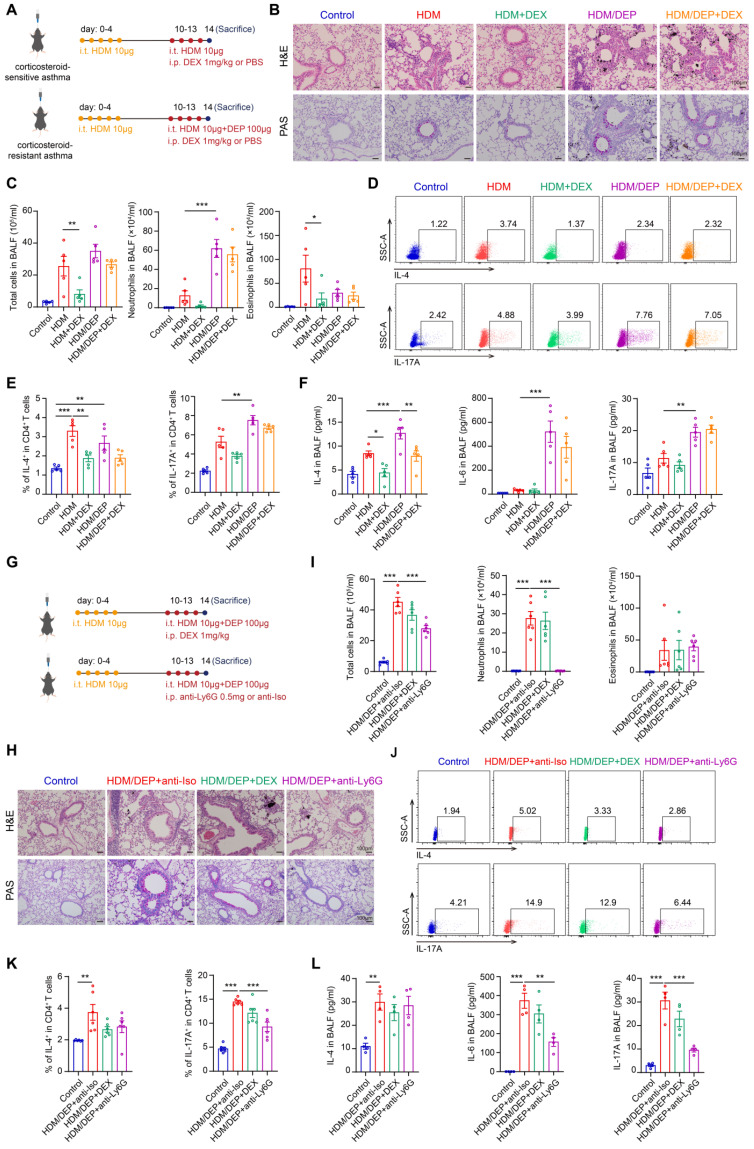
** DEP exposure results in corticosteroid-resistant allergic airway inflammation. (A)** HDM-induced asthma and HDM/DEP-induced corticosteroid-resistant asthma mouse models were established. DEX was administrated intraperitoneally. **(B)** Representative H&E and PAS staining pictures of lung tissues (n = 5 mice per group). Scale bar, 100 μm. **(C)** Total cell, neutrophil, and eosinophil counts in BALF measured by flow cytometry (n = 5 mice per group). **(D and E)** The percentage of IL-4^+^ (Th2) and IL-17A^+^ (Th17) cells in CD4^+^ T cells (gated on Live, CD45^+^CD3^+^CD4^+^) measured using flow cytometry (n = 5 mice per group). **(F)** Levels of inflammatory cytokines in BALF measured by ELISA (n = 5 mice per group). **(G)** For neutrophil depletion in HDM/DEP-induced corticosteroid-resistant asthma, 0.5 mg of anti-Ly6G antibody or the isotype control antibody was administered intraperitoneally on days 10-13, 1 hour prior to each challenge. **(H)** Representative H&E and PAS staining pictures of lung tissues (n = 6 mice per group). Scale bar, 100 μm. **(I)** Total cell, neutrophil, and eosinophil counts in BALF measured by flow cytometry (n = 6 mice per group). **(J and K)** The percentage of IL-4^+^ (Th2) and IL-17A^+^ (Th17) cells in CD4^+^ T cells (gated on Live, CD45^+^CD3^+^CD4^+^) measured using flow cytometry (n = 6 mice per group). **(L)** Levels of inflammatory cytokines in BALF measured by ELISA (n = 4 mice per group). Data are presented as means ± SEM. ^*^P < 0.05, ^**^P < 0.01,^ ***^P < 0.001. i.t., intratracheally; i.p., intraperitoneally; HDM, house dust mite; DEP, diesel exhaust particles; DEX, dexamethasone; BALF, bronchoalveolar lavage fluid.

We established asthma models using HDM alone as well as in combination with DEP to investigate their effects on airway inflammation. The impact of DEX was examined by intraperitoneal injection in both the HDM and HDM/DEP mouse models (Figure 1A). The HDM/DEP group exhibited a significant increase in inflammatory cell infiltration and mucus secretion around the airways compared to the HDM group, with distinct DEP deposits observed in areas of inflammatory infiltration. Flow cytometry was employed to elucidate the effects of DEP exposure on the recruitment of eosinophils and neutrophils in BALF. Following DEP exposure, we observed a marked increase in neutrophil infiltration in the BALF as compared to the HDM group. The airway inflammation, mucus secretion, and infiltration of eosinophils in BALF were effectively suppressed by DEX in the HDM group. In contrast, DEX administration in HDM/DEP-exposed asthmatic mice did not lead to a significant alleviation of airway inflammation, as evidenced by the lack of marked suppression of peribronchial infiltrates, mucus hypersecretion, and inflammatory cell infiltration in BALF (Figure 1B-C).

The Th17 cells-mediated immune response plays an important role in corticosteroid-resistant neutrophilic asthma 23. We evaluated the Th2 and Th17 responses by measuring the recruitment of Th2 and Th17 cells in the lung tissues as well as the levels of cytokines such as IL-4, IL-6, and IL-17A in BALF. In the HDM group, there was an increase in Th2 cells and a corresponding elevation in IL-4 levels. HDM/DEP group demonstrated a more pronounced Th17 response, as evidenced by a significant increase in Th17 cells in the lung tissues, and enhanced IL-6 and IL-17A levels in the BALF (Figure 1D-F). While DEX successfully reduced the levels of IL-4 in both HDM and HDM/DEP groups, it was ineffective in the HDM/DEP group in inhibiting the increased Th2 and Th17 cells as well as the production of neutrophil-related cytokines IL-6 and IL-17A (Figure 1D-F). In summary, both mouse models exhibited a comparable Th2 response, whereas the HDM/DEP group exhibited additional characteristics of the Th17 response, neutrophilic inflammation, and corticosteroid-resistance.

Neutrophils and Th17 cells may play a critical role in the exacerbation of asthma and the development of corticosteroid resistance. To investigate the inflammatory contributions by neutrophils, we administered a neutrophil-depleting antibody targeting Ly6G to deplete neutrophils in HDM/DEP model (Figure 1G). The administration of anti-Ly6G effectively diminished the neutrophils population in BALF and significantly alleviated airway inflammation and mucus secretion (Figure 1H-I). The depletion of neutrophils mainly affected the Th17 response, which was characterized by a significant reduction of Th17 cells in the lung tissues and decreased levels of IL-6 and IL-17A in BALF. In contrast, eosinophilic inflammation and Th2-mediated immunity remained largely unaffected (Figure 1J-L).

To further delineate the role of Th17 cells in corticosteroid-resistant asthma, we administered an anti-IL-17A antibody to inhibit Th17 response in HDM/DEP-exposed mice (Figure S2A). The depletion of Th17 cells ameliorated airway inflammation as indicated by fewer peribronchial infiltrates, reduced goblet cell hyperplasia, and a decrease in neutrophils in BALF (Figure S2B-C). Anti-IL-17A antibody significantly suppressed the activation and recruitment of Th17 cells or the secretion of IL-6 and IL-17A in BALF, while showing no discernible effects on eosinophilic inflammation and Th2 response (Figure S2D-F). In summary, our findings suggested that both neutrophils and Th17 were contributing factors to airway inflammation in corticosteroid-resistant asthma.

### DEP enhances ITA production by inducing metabolic reprogramming in neutrophils

**Figure 2:**
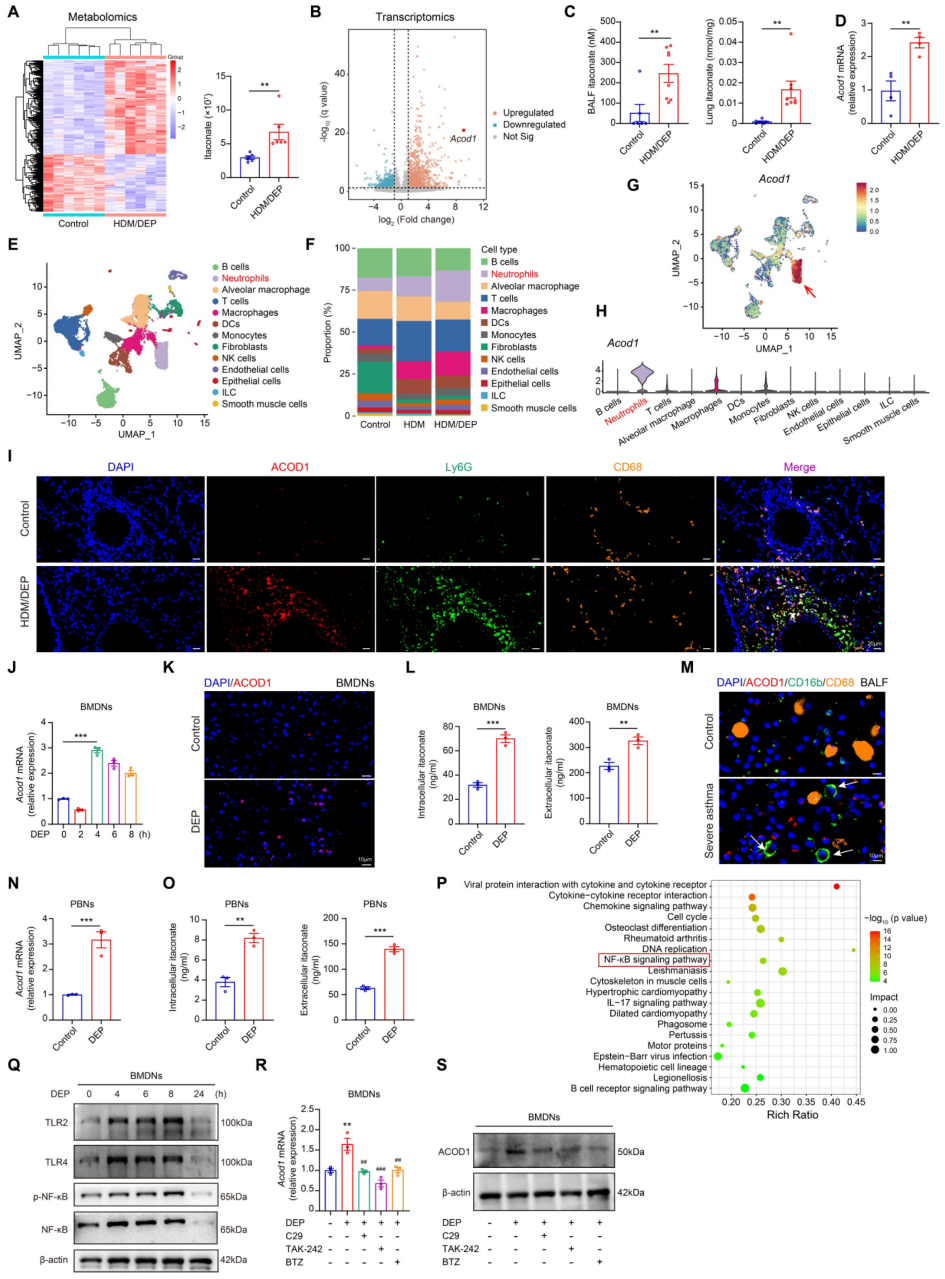
** DEP increases ITA production by inducing metabolic reprogramming in neutrophils. (A)** Heatmap of differentially accumulated metabolites between control and HDM/DEP groups, and the change of ITA between control and HDM/DEP groups in the sequencing data. **(B)** Volcano plot of the differentially expressed genes between the control and HDM/DEP groups. **(C)** The levels of ITA measured by LC/MS in BALF and lung tissues of HDM/DEP-induced asthma (n = 6-8 mice per group). **(D)** Real-time qPCR analysis of *Acod1* mRNA expression in the lung tissues of HDM/DEP-induced asthma (n = 4 mice per group). **(E)** Uniform manifold approximation and projection (UMAP) plot showing 13 clusters of cells in the lung tissues. **(F)** The percentage of the 13 clusters of cells in the lung tissues of con, HDM, and HDM/DEP groups. **(G)** The expression of *Acod1* in different cell clusters. **(H)** Violin plot of the expression of *Acod1* in different cell clusters. **(I)** Representative immunofluorescence staining of ACOD1 in the lung tissues of DEP-exposed asthmatic mice. Ly6G was used to mark neutrophils, and CD68 to mark macrophages. ACOD1 was co-localized with neutrophils. Scale bar, 20 μm. **(J)** Real-time qPCR analysis of *Acod1* mRNA expression in BMDNs treated with DEP. **(K)** Representative immunofluorescence staining of ACOD1 in BMDNs treated with DEP. Scale bar, 10 μm. **(L)** Intracellular and extracellular levels of ITA measured by LC/MS in DEP-stimulated BMDNs. **(M)** Representative immunofluorescence staining of ACOD1 in cell pellets of BALF from healthy control and patients with severe asthma. Neutrophils were marked with CD16b, and macrophages were marked with CD68. ACOD1 was co-localized with neutrophils. Scale bar, 10 μm. **(N)** Real-time qPCR analysis of *ACOD1* mRNA expression in PBNs stimulated with DEP. **(O)** Intracellular and extracellular levels of ITA measured by LC/MS in DEP-stimulated PBNs. **(P)** Integrated analysis of metabolomics and transcriptomics. **(Q)** Western blot analysis of TLRs and NF-κB signaling pathways in DEP-treated BMDNs. **(R)** Real-time qPCR analysis of *Acod1* mRNA expression in DEP-stimulated BMDNs pre-treated with TLR2 inhibitor (C29), TLR4 inhibitor (TAK-242), and NF-κB inhibitor (BTZ). ^**^P < 0.01, compared with the control group; ^##^*P* < 0.01, ^###^*P* < 0.001, compared with the DEP group. **(S)** Western blot analysis of ACOD1 expression in DEP-stimulated BMDNs pre-treated with TLR2 inhibitor (C29), TLR4 inhibitor (TAK-242), and NF-κB inhibitor (BTZ). Data are presented as means ± SEM. ^**^P < 0.01,^ ***^P < 0.001. HDM, house dust mite; DEP, diesel exhaust particles; LC/MS, Liquid Chromatography-Mass Spectrometry; BALF, bronchoalveolar lavage fluid; ACOD1, aconitate decarboxylase 1; BMDNs, bone marrow-derived neutrophils; PBNs, peripheral blood neutrophils; TLR, toll-like receptor; BTZ, Bortezomib.

To identify the key metabolic and transcriptomic alterations caused by DEP exposure in asthma, we performed untargeted metabolomic analysis and RNA-sequencing in HDM or HDM/DEP-induced asthma models and the control group. Our analysis revealed a significant metabolic change in the HDM/DEP group compared to the control group, with 355 upregulated DAMs and 217 downregulated DAMs (Figure 2A). When compared with the HDM group, the HDM/DEP group exhibited 37 DAMs, of which 24 were upregulated and 13 were downregulated (Figure S3A). Notably, ITA was markedly upregulated in the HDM/DEP group in comparison with both the control and HDM groups (Figure 2A, Figure S3A). Transcriptomic analysis also revealed that *Acod1*, the key enzyme responsible for ITA synthesis, was among the top 10 upregulated DEGs between the HDM/DEP group and control (Figure 2B). We further validated the production of ITA by quantifying ITA levels in BALF and lung tissues via LC/MS, which demonstrated a significant increase in ITA levels in both BALF and lung tissues of HDM/DEP-induced corticosteroid-resistant asthma (Figure 2C). Correspondingly, the mRNA expression of *Acod1* in the lung tissues of asthmatic mice exposed to DEP was also significantly increased, consistent with our transcriptomic findings (Figure 2D).

We performed scRNA-seq to identify the cell type responsible for ITA production during DEP-induced airway inflammation. A total of 13 cell clusters were identified including macrophages, T cells, neutrophils, and DC cells by scRNA-seq (Figure 2E). In line with our initial findings, there was a marked increase in neutrophils in the lung tissues of DEP-exposed asthmatic mice, with neutrophils and T cells representing the top two most abundant cell types (Figure 2F). Notably, neutrophils possessed the highest levels of *Acod1* expression within these populations (Figure 2G-H). Considering that previous studies reported that ITA is one of the most highly induced metabolites in activated macrophages 18, we used immunofluorescence to verify the cellular localization of ACOD1 in our model. Our findings demonstrated that ACOD1 was upregulated in Ly6G^+^ neutrophils in lung tissues of HDM/DEP asthmatic mice (Figure 2I), suggesting that neutrophils predominantly expressed ACOD1 and were the primary source of ITA synthesis in HDM/DEP-induced asthma.

To gain mechanistic insights into ITA metabolism in neutrophils, mouse BMDNs were isolated (Figure S3B) and treated with DEP for 2, 4, 6, or 8 hours. *Acod1* gene expression was significantly upregulated by DEP in BMDNs, reaching maximal expression level at 4 hours after treatment (Figure 2J). The upregulation of ACOD1 protein in DEP-treated BMDNs was also confirmed by immunofluorescence (Figure 2K). DEP treatment also caused a significant increase in both the intracellular and extracellular ITA (Figure 2L). Furthermore, we also confirmed ITA production in human samples. The expression of ACOD1 in cell pellets of BALF from healthy controls and patients with severe asthma was analyzed by immunofluorescence. ACOD1 was expressed in CD16b^+^ neutrophils and was significantly upregulated in neutrophils from the BALF of severe asthma patients compared to healthy controls (Figure 2M). PBNs were also extracted in our study (Figure S3C). RT-qPCR indicated that the expression of *ACOD1* in DEP-stimulated PBNs was increased (Figure 2N). Consistent with our findings in BMDNs, the intracellular and extracellular concentration of ITA in PBNs was increased after DEP treatment (Figure 2O). In summary, our results suggested that DEP activated ACOD1 expression and promoted ITA synthesis in neutrophils.

To elucidate the mechanism underlying DEP-induced *Acod1* expression, we conducted a conjoint analysis of metabolomics and transcriptomics data utilizing MetaboAnalyst 5.0. The results showed that cytokine-cytokine receptor interaction, NF-κB signaling pathway, and IL-17 signaling pathway were among the top 20 significantly changed pathways (Figure 2P). We examined the alterations of the NF-κB signaling pathways in BMDNs stimulated with DEP. The results revealed that DEP exposure promoted the expression of TLR2 and TLR4, and the activation of the NF-κB pathway (Figure 2Q, Figure S3D-H). Furthermore, the intervention of pharmacological inhibitors targeting TLR2, TLR4, and NF-κB pathways resulted in a significant suppression of ACOD1 expression (Figure 2R-S, Figure S3I). The findings indicated that TLR/NF-κB axis contributed to the expression of ACOD1 in neutrophils in response to DEP.

### ACOD1/ITA alleviates airway inflammation in corticosteroid-resistant asthma

**Figure 3:**
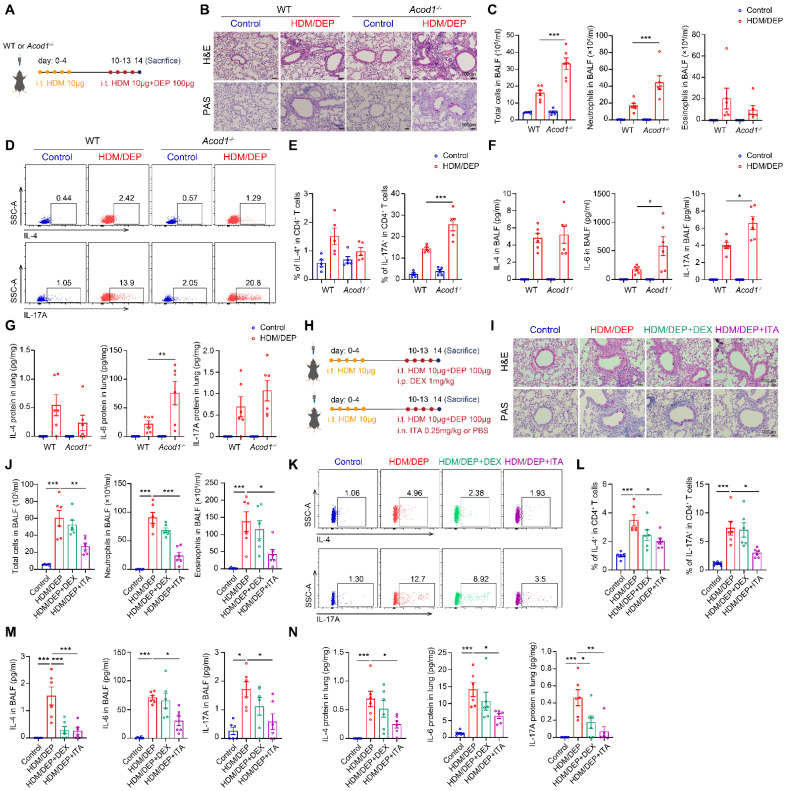
** ACOD1/ITA alleviates airway inflammation in corticosteroid-resistant asthma. (A)** WT or *Acod1^-/-^* mice were sensitized with HDM, followed by challenge with HDM and DEP. **(B)** Representative H&E and PAS staining pictures of lung tissues (n = 6 mice per group). Scale bar, 100 μm. **(C)** Total cell, neutrophil, and eosinophil counts in BALF measured by flow cytometry (n = 6 mice per group). **(D and E)** The percentage of IL-4^+^ (Th2) and IL-17A^+^ (Th17) cells in CD4^+^ T cells (gated on Live, CD45^+^CD3^+^CD4^+^) measured using flow cytometry (n = 5 mice per group). **(F)** Levels of inflammatory cytokines in BALF measured by using a cytometric bead array (n = 6 mice per group). **(G)** Levels of inflammatory cytokines in lung homogenates measured by using a cytometric bead array (n = 6 mice per group). **(H)** HDM/DEP-exposed asthmatic mice were established. DEX or ITA was administered 1 hour before HDM and DEP challenge. **(I)** Representative H&E and PAS staining pictures of lung tissues (n = 6 mice per group). Scale bar, 100 μm. **(J)** Total cell, neutrophil, and eosinophil counts in BALF measured by flow cytometry (n = 6 mice per group). **(K and L)** The percentage of IL-4^+^ (Th2) and IL-17A^+^ (Th17) cells in CD4^+^ T cells (gated on Live, CD45^+^CD3^+^CD4^+^) measured using flow cytometry (n = 6 mice per group). **(M)** Levels of inflammatory cytokines in BALF measured by using a cytometric bead array (n = 6 mice per group). **(N)** Levels of inflammatory cytokines in lung homogenates measured by using a cytometric bead array (n = 6 mice per group). Data are presented as means ± SEM. ^*^P < 0.05, ^**^P < 0.01,^ ***^P < 0.001. i.t., intratracheally; i.p., intraperitoneally; i.n. intranasally; HDM, house dust mite; DEP, diesel exhaust particles; ACOD1, aconitate decarboxylase 1; DEX, dexamethasone; ITA, itaconate; BALF, bronchoalveolar lavage fluid.

We utilized wild-type (WT) and *Acod1*^-/-^ mice to investigate the role of ITA in HDM/DEP-induced corticosteroid-resistant asthma (Figure 3A). Histology analysis demonstrated increased peribronchial inflammatory cell infiltration and airway mucus secretion in *Acod1^-/-^* mice compared to WT asthmatic mice exposed to DEP (Figure 3B). Flow cytometry was used to detect the changes in eosinophils and neutrophils in BALF. We found that *Acod1* knockout resulted in a significant increase of neutrophils in BALF, indicating airway inflammation further shifted towards a neutrophilic phenotype (Figure 3C). Regarding Th2 and Th17 immune responses, genetic ablation of *Acod1* did not significantly alter Th2 cell populations but substantially increased Th17 cells recruitment to lung tissues following DEP challenge (Figure 3D-E). Cytokine analysis further confirmed that *Acod1* knockout promoted the expression and secretion of IL-6 and IL-17A, while leaving IL-4 levels unchanged (Figure 3F-G). Collectively, these findings demonstrated that *Acod1*^-/-^ mice exhibited exacerbated neutrophilic airway inflammation and promoted a Th17-mediated immune response in the HDM/DEP-induced asthma, indicating that ITA was a negative regulator of corticosteroid-resistant asthma.

Next, we evaluated whether supplementation of ITA or its derivative 4-OI alleviated asthma symptoms. ITA or 4-OI were administered 1 hour before HDM and DEP challenge (Figure 3H, Figure S4A). Histopathological analysis of lung tissue sections revealed that both ITA and 4-OI significantly attenuated airway inflammation and mucus hypersecretion, while DEX demonstrated minimal therapeutic effect (Figure 3I, Figure S4B). Additionally, ITA and 4-OI significantly inhibited the infiltration of eosinophils and neutrophils in BALF, compared with the control and DEX treatment group (Figure 3J, Figure S4C). Flow cytometry of lung tissues further demonstrated that DEX failed to inhibit the Th2 and Th17 responses, whereas ITA and 4-OI significantly attenuated the activation and recruitment of Th2 and Th17 cells in the lung tissues (Figure 3K-L, Figure S4D-E). Cytokine analysis indicated that ITA or 4-OI significantly suppressed elevated levels of IL-4, IL-6, and IL-17A in BALF and lung tissues (Figure 3M-N, Figure S4F-G). Collectively, our findings supported that ITA exerted a protective role against airway inflammation in corticosteroid-resistant asthma.

We also validated the anti-inflammatory effect of ITA and ACOD1 in another model of corticosteroid-resistant asthma induced by HDM and LPS (Figure S5A). In the HDM/LPS model, neutrophil was also the major cell type expressing ACOD1 (Figure S5B). Similar to our observation from the HDM/DEP model, *Acod1* knockout in the HDM/LPS model also exacerbated airway inflammation and mucus secretion (Figure S5C), as well as increased neutrophilic airway inflammation and a Th17-mediated immune response (Figure S5D-G). Notably, exogenous supplementation of ITA was found to attenuate airway inflammation in HDM/LPS model, evidenced by decreased peribronchial inflammatory cells infiltration and lower levels of eosinophils and neutrophils infiltration in BALF (Figure S6A-C). The recruitment of Th2 and Th17 cells in the lung tissues, as well as the levels of IL-4, IL-6, and IL-17A in BALF and lung tissues, were also inhibited by ITA in the HDM/LPS model (Figure S6D-F). In summary, these results demonstrate the therapeutic potential of the ACOD1/ITA axis for managing inflammation in corticosteroid-resistant asthma.

### ACOD1/ITA inhibits DEP-induced inflammatory responses in neutrophils and Th17 cell differentiation *in vitro*

**Figure 4:**
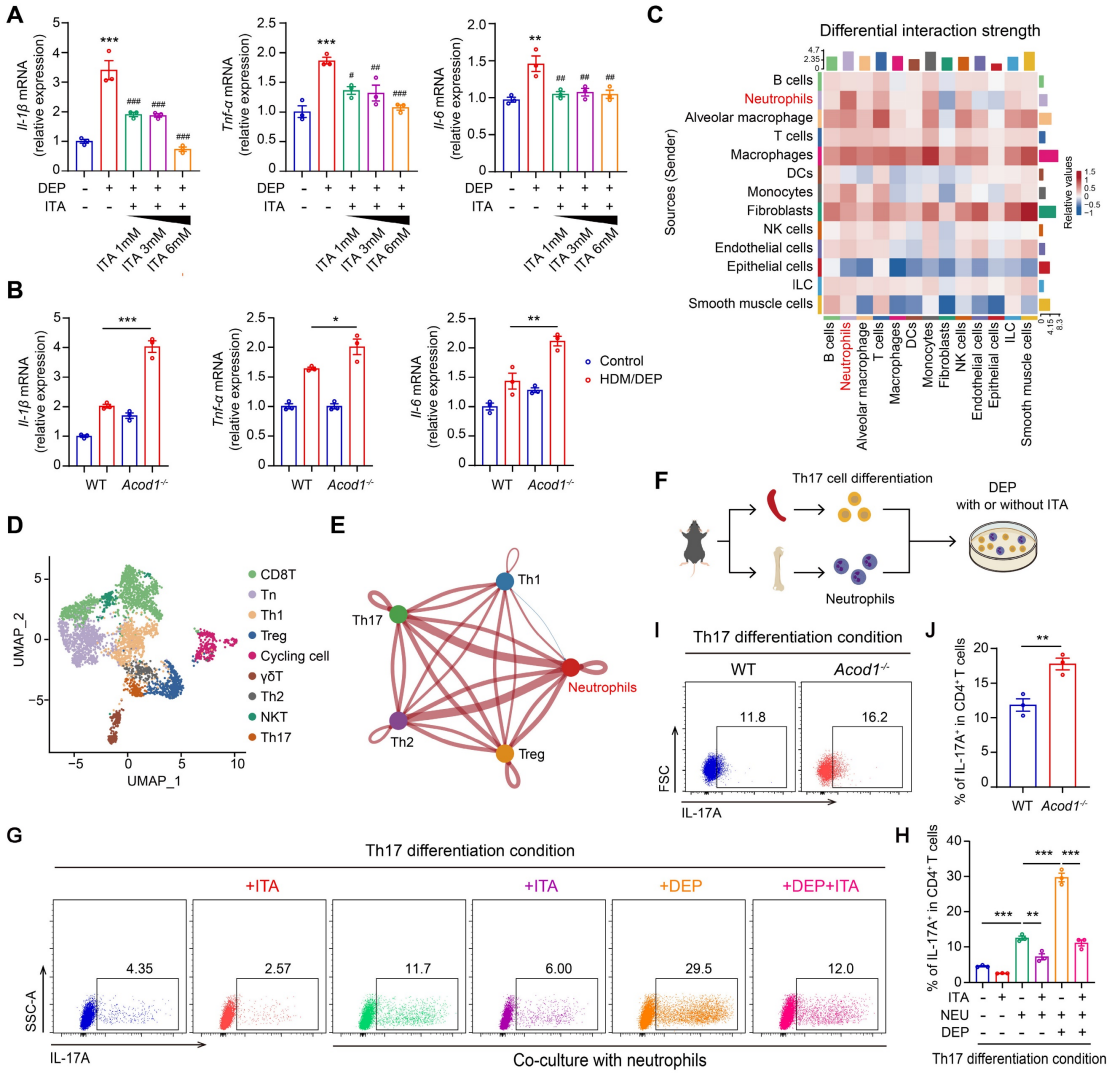
** ACOD1/ITA inhibits DEP-induced inflammatory responses in neutrophils and Th17 cell differentiation *in vitro*. (A)** Real-time qPCR analysis of inflammatory cytokines (*Il-1β*, *Tnf-α*, and *Il-6*) in BMDNs stimulated with DEP and treated with ITA. ^**^P < 0.01,^ ***^P < 0.001, compared with the control group. ^#^*P* < 0.05, ^##^*P* < 0.01, ^###^*P* < 0.001, compared with the DEP group. **(B)** Real-time qPCR analysis of inflammatory cytokines (*Il-1β*, *Tnf-α*, and *Il-6*) in DEP-stimulated BMDNs from WT or *Acod1^-/-^* mice. **(C)** Cellchat analysis of intercellular communication networks from scRNA-seq data. **(D)** Identification of T cell clusters. **(E)** The intercellular communication between neutrophils and Th1, Th2, Th17, and Treg cells. **(F)** Naive CD4^+^ T cells from mouse spleen were co-cultured with BMDNs in U-bottom 96-well plates in Th17 differentiation condition. After 6 hours, ITA (6 mM) was added to the appropriate wells. One hour later, cells were stimulated with DEP (100 μg/mL). After 72-96 h, cells were harvested for flow cytometry. **(G and H)** The percentage of IL-17A^+^ (Th17) cells in CD4^+^ T cells measured using flow cytometry to detect the effect of ITA on Th17 cell differentiation under different stimulation conditions. **(I and J)** The percentage of IL-17A^+^ (Th17) cells in CD4^+^ T cells measured using flow cytometry under the co-culture system of naive CD4^+^ T cells from WT mice and BMDNs from WT or *Acod1^-/-^* mice. Data are presented as means ± SEM. ^*^P < 0.05, ^**^P < 0.01,^ ***^P < 0.001. DEP, diesel exhaust particles; BMDNs, bone marrow-derived neutrophils; ITA, itaconate; NEU, neutrophils; ACOD1, aconitate decarboxylase 1.

Given that neutrophils serve as a predominant source of ITA, we first focused on the effects of ITA on the intrinsic inflammatory responses of neutrophils. We found that DEP promoted the expression of inflammatory cytokines *Il-1β*, *Tnf-α*, and *Il-6* in BMDNs, while exogenous ITA supplemented in culture medium inhibited the expression of DEP-induced inflammatory cytokines in a concentration-dependent manner (Figure 4A). We further explored the impact of *Acod1* knockout on neutrophils. BMDNs were isolated from WT and *Acod1*^-/-^ mice and subsequently stimulated with DEP. *Acod1*^-/-^ neutrophils demonstrated an amplified inflammatory response compared to WT neutrophils, as evidenced by the increased expression of *Il-1β*, *Tnf-α,* and *Il-6* (Figure 4B). We further characterized the inflammatory response of *Acod1*^-/-^ neutrophils to LPS stimulation. *Acod1* expression was upregulated in response to LPS stimulation in BMDNs (Figure S7A). Consistent with results from DEP-stimulated BMDNs, genetic deletion of *Acod1* exacerbated inflammatory responses, whereas exogenous ITA administration exerted potent anti-inflammatory effects (Figure S7B-C). In addition, 4-OI also inhibited the inflammatory responses in DEP or LPS-stimulated BMDNs (Figure S8A-B).

We then employed Cellchat to analyze intercellular communication networks from scRNA-seq data. Our analysis revealed distinct communication relationships between neutrophils and T cells (Figure 4C). Unsupervised hierarchical clustering and visualization with t-distributed stochastic neighbor embedding (t-SNE) identified nine clusters of T cells (Figure S9A). Helper T cells (Th cells) and regulatory T cells (Treg) are regarded as central players in the pathogenesis of asthma 24. We classified T cells into subpopulations based on characteristic gene markers (Figure 4D). Among the CD4^+^ clusters (Figure S9B), cells in cluster 3 expressed transcripts for *Cxcr3*, *Tbx21*, *Ifn-γ*, *Csf2*, *Il3*, and *Il2*, identified as Th1 cells (Figure S9C). The upper half of cluster 7 cells were highly enriched for *Gata3*, *Il13*, *Il5*, *Il1rl1*, and *Stat6*, indicative of a Th2 cell phenotype (Figure S9D). The lower half of cluster 7 expressed *Il23r*, *Ccr6*, *Ctsh*, *Il17f*, and *Il17a*, indicative of a Th17 cell phenotype (Figure S9E). Cells in cluster 5 most resembled classical Treg cells, since they expressed *Foxp3*, *Ctla4*, *Il10*, *Il2ra*, and *Klrg1* (Figure S9F). Subsequently, we delved into the communication between neutrophils and the identified Th subpopulations, including Th1, Th2, Th17, and Treg cells. The results revealed communication relationships between neutrophils and both Th2 and Th17 cells (Figure 4E). In our model, we focused primarily on the interactions between neutrophils and Th17 cells, as ITA and genetic ablation of *Acod1* did not significantly alter Th2 cell populations.

We isolated naive CD4^+^ T cells from the spleen, differentiated naive T cells into Th17 and ITA was added to the medium. Notably, ITA significantly suppressed IL-17A production in CD4^+^ T cells under Th17-polarization conditions (Figure S10A-C), indicating a direct inhibitory effect of ITA on Th17 cell differentiation. Subsequently, Th17 was co-cultured with neutrophils. After a 6-hour initial cell plating, ITA was added to the medium, followed by the addition of DEP 1 hour later (Figure 4F). Following 72-96 hours of culture, neutrophils significantly promoted Th17 cell differentiation, which was further increased with additional DEP treatment. Conversely, ITA treatment inhibited Th17 cell differentiation (Figure 4G-H). When neutrophils harvested from the bone marrow of WT or *Acod1^-/-^*mice were co-cultured with naive CD4^+^ T cells, *Acod1^-/-^* neutrophils exhibited a stronger ability to promote Th17 cell differentiation than WT neutrophils (Figure 4I-J). Our results suggested that ACOD1/ITA inhibited DEP-induced inflammatory responses in neutrophils and Th17 cell differentiation.

### ACOD1/ITA inhibits the formation of NETs to attenuate airway inflammation in corticosteroid-resistant asthma

**Figure 5:**
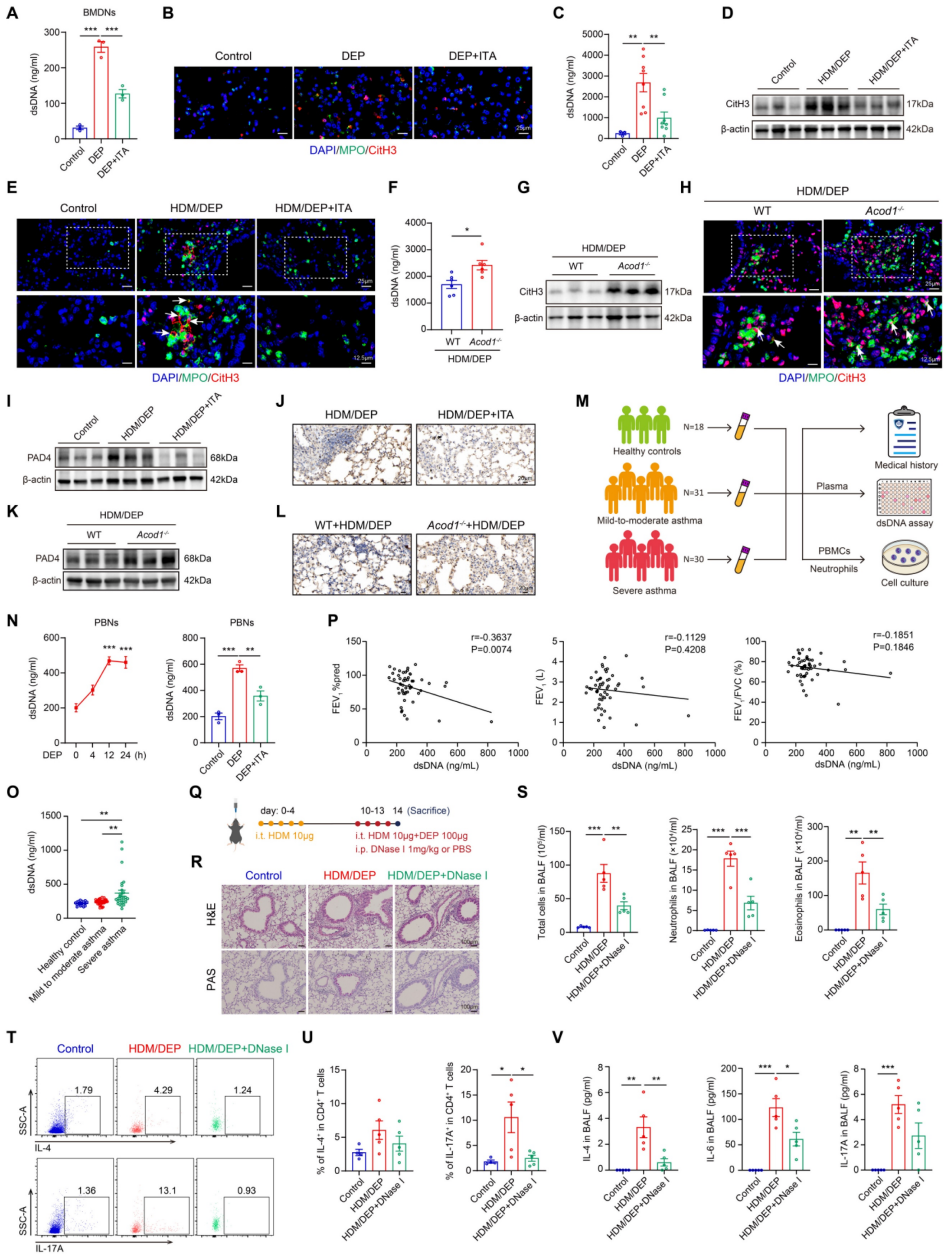
** ACOD1/ITA inhibits the formation of NETs to attenuate airway inflammation in corticosteroid-resistant asthma. (A)** The concentration of dsDNA in BMDNs stimulated with DEP and treated with ITA. **(B)** Representative immunofluorescence staining of CitH3 and MPO in BMDNs stimulated with DEP and treated with ITA. Scale bar, 25 μm. **(C)** The concentration of dsDNA in BALF of HDM/DEP-induced asthma with or without treatment of ITA. **(D)** Western blot analysis of CitH3 in the lung tissues of HDM/DEP-induced asthma with or without treatment of ITA. **(E)** Representative immunofluorescence staining of CitH3 and MPO in the lung tissues of HDM/DEP-induced asthma with or without treatment of ITA. The arrows indicate the colocalization of CitH3 and MPO. **(F)** The concentration of dsDNA in BALF of HDM/DEP-induced asthma in WT or *Acod1^-/-^* mice. **(G)** Western blot analysis of CitH3 in the lung tissues of HDM/DEP-induced asthma in WT or *Acod1^-/-^* mice. **(H)** Representative immunofluorescence staining of CitH3 and MPO in the lung tissues of HDM/DEP-induced asthma in WT or *Acod1^-/-^*mice. The arrows indicate the colocalization of CitH3 and MPO. **(I)** Western blot analysis of PAD4 in the lung tissues of HDM/DEP-induced asthma with or without treatment of ITA. **(J)** Immunohistochemical staining of PAD4 in lung tissue of HDM/DEP mice and treated with or without ITA. Scale bar, 20μm. **(K)** Western blot analysis of PAD4 in the lung tissues of HDM/DEP-induced asthma in WT or *Acod1^-/-^* mice. **(L)** Immunohistochemical staining of PAD4 in lung tissue of HDM/DEP mice in WT or *Acod1^-/-^* mice. Scale bar, 20μm. **(M)** Human blood samples were collected. Plasma, PBMCs, and neutrophils were isolated for further study. **(N)** The concentration of dsDNA in supernatant of PBNs stimulated with DEP for different time points and treated with ITA.^ ***^P < 0.001, compared with the control group. **(O)** The concentration of dsDNA in plasma of healthy control, mild-to-moderate asthma patients, and severe asthma patients. **(P)** Correlation analysis of dsDNA and lung function index FEV_1_%pred, FEV_1_, and FEV_1_/FVC. **(Q)** HDM/DEP-induced asthmatic mice were established. NETs inhibitor DNase I was administered 1 hour before HDM and DEP challenge. **(R)** Representative H&E and PAS staining pictures of lung tissues (n = 5 mice per group). Scale bar, 100 μm. **(S)** Total cell, neutrophil, and eosinophil counts in BALF measured by flow cytometry (n = 5 mice per group). **(T-U)** The percentage of IL-4^+^ (Th2) and IL-17A^+^ (Th17) cells in CD4^+^ T cells (gated on Live, CD45^+^CD3^+^CD4^+^) measured using flow cytometry (n = 4-5 mice per group). **(V)** Levels of inflammatory cytokines in BALF measured by using a cytometric bead array (n = 5 mice per group). Data are presented as means ± SEM. ^*^P < 0.05, ^**^P < 0.01,^ ***^P < 0.001. CitH3, citrulline histone H3; dsDNA, double-stranded DNA; i.t., intratracheally; i.p., intraperitoneally; HDM, house dust mite; DEP, diesel exhaust particles; BALF, bronchoalveolar lavage fluid; BMDNs, bone marrow-derived neutrophils; ITA, itaconate; ACOD1, aconitate decarboxylase 1; PBMCs, peripheral blood mononuclear cells; PBNs, peripheral blood neutrophils; PAD4, peptidylarginine deiminase 4; NETs, neutrophil extracellular traps.

We hypothesized that ITA inhibited the ability of neutrophils to promote Th17 cell differentiation by suppressing the formation of NETs in corticosteroid-resistant asthma. To test this hypothesis, we evaluated the effects of ITA on NETs formation both *in vitro* and *in vivo*. BMDNs were stimulated with DEP and treated with ITA. Detection of the release of dsDNA and immunofluorescence staining of CitH3 in MPO^+^ BMDNs showed that DEP-induced NETs were significantly inhibited by ITA (Figure 5A-B). Similarly, ITA inhibited NETs in HDM/DEP-induced asthmatic mice, as evidenced by reduced concentration of dsDNA in BALF, and decreased CitH3 in neutrophils in the lung tissues (Figure 5C-E, Figure S10D). Conversely, *Acod1^-/-^* mice displayed a significant enhancement in NETs formation, with elevated dsDNA concentration in BALF and increased CitH3 in the lung tissues (Figure 5F-H, Figure S10E). PAD4 plays a pivotal role in the formation of NETs 25. We found that treatment of ITA inhibited PAD4 expression in the lung tissues of HDM/DEP-induced asthma, while knockout of *Acod1* promoted the expression of PAD4 (Figure 5I-L, Figure S10F-G). Overall, the findings demonstrated that ACOD1/ITA suppressed NETs formation in corticosteroid-resistant asthma.

Human blood samples were also collected to identify the role of NETs in asthma (Figure 5M, Table S2). PBNs were stimulated with DEP, treated with or without ITA. Then, the supernatant was collected and the concentration of NETs was measured. DEP promoted NETs formation in a time-dependent manner, while treatment of ITA significantly inhibited DEP-induced NETs formation in PBNs (Figure 5N). To clarify the association between NETs and severe asthma in human patients, we quantified the concentrations of dsDNA in the plasma of healthy controls, patients with mild-to-moderate asthma, and those with severe asthma. Severe asthma patients had higher levels of dsDNA in the plasma than those in mild-to-moderate asthma patients and healthy controls (Figure 5O). Moreover, plasma dsDNA levels showed a significant negative correlation with the lung function index, FEV_1_%pred, in asthmatic patients. While dsDNA levels also displayed negative correlations with FEV_1_ and FEV_1_/FVC, these correlations did not achieve statistical significance (Figure 5P). In summary, our results suggested that elevated NETs participated into the pathogenesis of severe asthma and were correlated with diminished lung function in patients.

We further explored the therapeutic potential of NETs inhibition to alleviate airway inflammation in corticosteroid-resistant asthma by administration of DNase I (Figure 5Q). Notably, DNase I significantly inhibited airway inflammation, mucus secretion, and the infiltration of eosinophils and neutrophils in BALF (Figure 5R-S). We also examined the impacts of NETs inhibition on the Th2 and Th17 responses. DNase I group showed marked decrease in Th17 cells in the lung tissues (Figure 5T-U). Further detection of the inflammatory cytokines showed that DNase I inhibited inflammatory cytokines in BALF (Figure 5V). The above results suggested that targeting NETs was a promising strategy to alleviate airway inflammation in HDM/DEP-induced corticosteroid-resistant asthma.

### ITA inhibits Th17 cell differentiation by suppressing the formation of NETs in corticosteroid-resistant asthma

**Figure 6:**
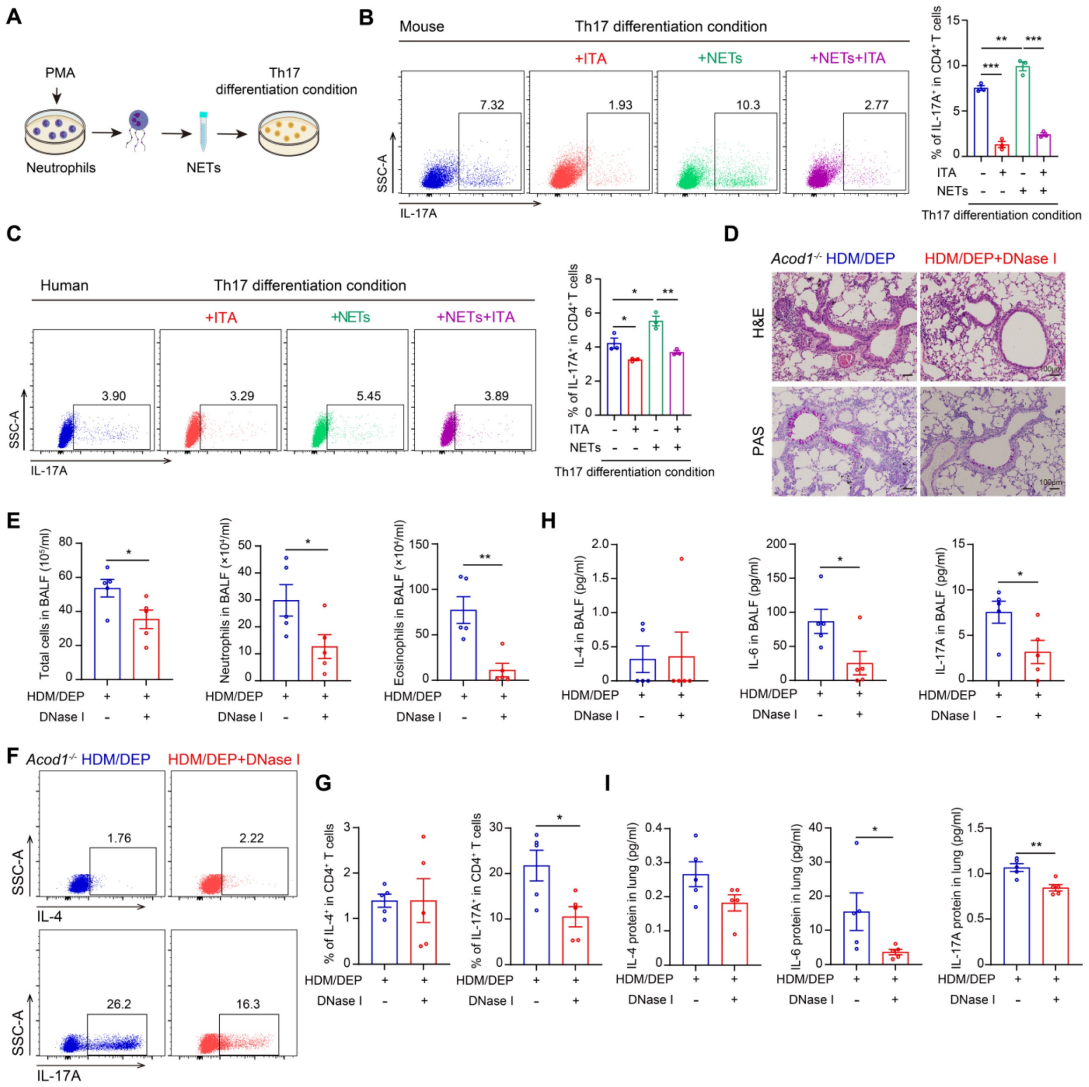
** ITA inhibits Th17 cell differentiation by suppressing the formation of NETs in corticosteroid-resistant asthma. (A)** Neutrophils were treated with PMA and the NETs were collected to stimulate naive CD4^+^ T cell *in vitro*. **(B)** Naive CD4^+^ T cells were isolated from mouse spleens and cultured under Th17 differentiation conditions. The cells were stimulated with NETs and treated with ITA. Flow cytometry was used to detect Th17 cell differentiation. **(C)** Naive CD4^+^ T cells were isolated from PBMCs and cultured under Th17 differentiation conditions. The cells were stimulated with NETs and treated with ITA. Flow cytometry was used to assess Th17 cell differentiation. **(D)** Representative H&E and PAS staining pictures of lung tissues (n = 5 mice per group). Scale bar, 100 μm. **(E)** Total cell, neutrophil, and eosinophil counts in BALF measured by flow cytometry (n = 5 mice per group). **(F and G)** The percentage of IL-4^+^ (Th2) and IL-17A^+^ (Th17) cells in CD4^+^ T cells (gated on Live, CD45^+^CD3^+^CD4^+^) measured using flow cytometry (n = 5 mice per group). **(H)** Levels of inflammatory cytokines in BALF measured by using a cytometric bead array (n = 5 mice per group). **(I)** Levels of inflammatory cytokines in lung homogenates measured by using a cytometric bead array (n = 5 mice per group). Data are presented as means ± SEM. ^*^P < 0.05, ^**^P < 0.01,^ ***^P < 0.001. PMA, phorbol 12-myristate 13-acetate; HDM, house dust mite; DEP, diesel exhaust particles; ACOD1, aconitate decarboxylase 1; BALF, bronchoalveolar lavage fluid; NETs, neutrophil extracellular traps; ITA, itaconate.

To elucidate the role of NETs in promoting Th17 cell differentiation, we directly stimulated naive CD4^+^ T cells with NETs in the presence or absence of ITA (Figure 6A). Our results showed that NETs promoted the differentiation of naive CD4^+^ T cells into Th17 cells *in vitro*, whereas ITA inhibited NETs-mediated Th17 cell differentiation (Figure 6B). The observed effects of ITA were reproducible in human naive CD4^+^ T cells isolated from PBMCs (Figure 6C), confirming cross-species conservation of this immunoregulatory mechanism.

To establish whether NET formation is required for mediating ITA-dependent regulation of Th17 cell differentiation, we treated HDM/DEP-induced *Acod1^-/-^* mice with NET inhibitor DNase I. Administration of DNase I alleviated airway inflammation and mucus secretion in *Acod1*^-/-^ HDM/DEP mice (Figure 6D), and also effectively suppressed the total cell counts and the number of eosinophils and neutrophils in the BALF (Figure 6E). Notably, the recruitment of Th17 cells in the lung tissues of HDM/DEP-induced corticosteroid-resistant asthma in *Acod1*^-/-^ mice was significantly inhibited by DNase I, paralleling a reduction in IL-6 and IL-17A in BALF and lung tissues (Figure 6F-I). Collectively, these results demonstrated that ITA decreased the formation of NETs from neutrophils, thereby further suppressing Th17 cell differentiation in HDM/DEP-induced corticosteroid-resistant asthma.

## Discussion

In this study, we identified ITA as a critical immunoregulatory metabolite in the pathogenesis of DEP-exacerbated asthma. When the respiratory tract was exposed to DEP, it could induce neutrophils filtration and the differentiation of Th17 cells, which collectively contribute to corticosteroid resistance in asthma triggered by HDM. A combination analysis of multi-omics and scRNA-seq revealed that ITA level was significantly elevated in corticosteroid-resistant asthma induced by HDM and DEP, with neutrophils identified as the primary cell type responsible for ITA production. DEP induced the production of ITA by activating the NF-κB signaling pathway. Notably, exogenous supplementation of ITA could alleviate HDM/DEP-induced airway inflammation, particularly non-T2 inflammation, and counteract corticosteroid resistance in asthma. Mechanistically, ITA decreased NETs secretion from neutrophils by inhibiting the expression of PAD4, thereby further suppressing Th17 cell differentiation. Our findings indicated that ITA played a critical role in suppressing neutrophilic airway inflammation in corticosteroid-resistant asthma, and highlighted potential therapeutic implications of ITA for patients suffering from corticosteroid-resistant asthma.

Corticosteroid resistance poses a significant challenge in the treatment and management of asthma patients in clinical practice. Based on T2 inflammation biomarkers, asthma can be primarily divided into two distinct endotypes: T2 asthma and non-T2 asthma. Notably, non-T2 asthma, particularly neutrophilic asthma, is more closely associated with corticosteroid resistance 26. Particulate matter has been established as a critical modulator of neutrophilic airway inflammation and corticosteroid resistance. In our previous study, we demonstrated that particulate matter promoted neutrophils infiltration and airway inflammation through the HDAC9-mediated epigenetic regulation of the DUSP9-MAPK signaling pathway 27. As a prototypical airborne particulate and environmental pollutant, DEP was found to induce neutrophilic airway inflammation and corticosteroid resistance in a murine model of asthma, and was also closely correlated with asthma control among children 6, 28. Neutrophils, activated by DEP exposure, have been implicated in promoting T2 and non-T2 inflammatory responses in asthma 29. Among non-T2 inflammation, the Th17 cell-mediated immune response has been reported to participate in corticosteroid-resistant neutrophilic asthma 23. Consistent with the established literatures, we found that neutralization of IL-17A could mitigate HDM/DEP-induced airway inflammation in models of corticosteroid-resistant asthma 6, 30. To identify the pivotal role of neutrophils in corticosteroid-resistant asthma, we employed the anti-Ly6G antibody to deplete neutrophils, which resulted in a notable attenuation of Th17 cell differentiation and airway inflammation in corticosteroid-resistant asthma. However, the potential modulator for the immune imbalance in corticosteroid-resistant asthma was not well elucidated. According to our analysis of multi-omics and scRNA-seq, metabolic reprogramming of neutrophils played an important role in regulating airway immune balance, which provided a new direction for treatment of corticosteroid resistance in asthma.

ITA is an endogenous metabolite that plays an important role in regulating immune and inflammatory homeostasis across multiple disease contexts 15. Evidence indicated that ITA levels increased in macrophages in response to various proinflammatory stimuli. For instance, LPS or PM exposure has been shown to promote abundant production of both intracellular and extracellular ITA in macrophages 31, 32. Neutrophils are another cell source of ITA, but research on neutrophil-derived ITA is relatively insufficient compared to that in macrophages. It was reported that staphylococcus aureus could increase the production of ITA in neutrophils 33. In the present study, we identified a significant elevation of ITA levels in HDM/DEP-induced corticosteroid-resistant asthma. Importantly, our findings indicated that ITA production predominantly originated from neutrophils rather than macrophages. Under pro-inflammatory conditions, ITA was rapidly induced in myeloid cells and exhibited its significant anti-inflammatory properties during periods of inflammation. This suggested that the body relied on metabolic reprogramming to establish a delicate balance of immunity and inflammation 34. ITA conferred protection against inflammatory responses induced by LPS through alkylated modification of the KEAP1-NRF2 pathway both *in vivo* and *in vitro* 19. Furthermore, ITA dampened LPS-induced inflammation in macrophages by inhibiting the enzymatic activity of succinate dehydrogenase and TET-family DNA dioxygenases 18, 21. However, ITA also showed an unfriendly effect under some specific pathological conditions. For example, neutrophil-derived ITA also played a dominant role in promoting viral infection 35. It is worth noting that ITA derived from different cells may exert entirely distinct effects in diseases. ITA exerted opposing effects on bone marrow-derived macrophages (BMDMs) and alveolar macrophages (AMs). It inhibited the expression of inflammatory factors in LPS-induced BMDMs, but had a pro-inflammatory effect in AMs 36. Distinct stimuli could influence the function of ITA. In BMDMs, ITA inhibited LPS-induced inflammation but failed to effectively alleviate PM-induced inflammation 32. Additionally, the role of ITA in diseases exhibited remarkable duality. Staphylococcus aureus could increase the production of ITA in neutrophils to protect the lung from excessive inflammation, but compromise bacterial clearance 33. In the present study, neutrophils-derived ITA could effectively mitigate airway inflammation in corticosteroid-resistant asthma induced by a combination of HDM and DEP and maintain the airway immune balance. These results presented the promising therapeutic potential of ITA in addressing corticosteroid-resistant asthma.

The mechanisms by which neutrophil-derived ITA exerts its effects remain unclear. NETs are web-like structures composed of genomic DNA, histones, and granule proteins that are released by activated neutrophils 37. The excessive formation of NETs contributed to tissue damage and inflammation 38. Previous studies have demonstrated that levels of NETs were markedly elevated in the lungs and sputum of severe asthma 39, 40. Furthermore, neutrophils isolated from the peripheral blood of corticosteroid-resistant asthma patients exhibited a significantly higher secretion of NETs upon stimulation with PMA compared to those from corticosteroid-sensitive asthma patients 14. Our findings also revealed that the abundance of NETs in the plasma of severe asthma patients was higher than in mild-to-moderate asthma patients or healthy controls. Furthermore, the level of NETs was positively associated with impaired lung function in severe asthma patients. The formation of NETs induced corticosteroid resistance in asthma via amplifying Th17 cells-mediated airway inflammation 41. In our study, supplementation with NETs increased Th17 cell differentiation *in vitro*. The potential mechanism might involve histones in NETs components directly promoting Th17 cell differentiation via activation of TLR2-STAT3 pathways in T cells 42, 43. Conversely, ITA could inhibit DEP-induced NETs formation in neutrophils, resulting in a significant reduction in Th17/IL-17-mediated airway inflammation. Moreover, our rescue experiment demonstrated that treatment with DNase I could restore Th17/IL-17-mediated airway inflammatory responses in *Acod1*^-/-^ HDM/DEP mice. PAD4 was the critical protein to mediate histone citrullination, which subsequently promoted NETs formation 44. In sepsis models, ACOD1/ITA enhanced the enzymatic activity of UBR5 through alkylation modification, promoting the K48-linked polyubiquitination and degradation of PAD4, thereby inhibiting NETosis 45. Notably, we demonstrated that ITA could decrease the expression of PAD4 in the lung tissues to inhibit NETs formation in HDM/DEP-induced asthma model. Besides, neutrophil-derived ITA could directly suppress Th17 cell differentiation in a paracrine manner. The regulatory mechanism appeared to involve ITA limiting the binding of RORγt to the IL-17A promoter 46. Previous research has demonstrated that TLR2/4 and its downstream NF-κB pathway could regulate ACOD1 expression in macrophages 16. Through the integrated analysis of multi-omics, our results demonstrated that DEP exposure enhanced ACOD1 expression in neutrophils through activation of the TLRs/NF-κB signaling pathway.

In conclusion, our study demonstrated that exposure to DEP could induce neutrophilic airway inflammation and corticosteroid resistance in asthma. Neutrophils acted as a dual modulator in corticosteroid-resistant asthma by producing ITA via the NF-κB signaling pathway. They released NETs, which promoted Th17/IL-17 responses associated with corticosteroid resistance. At the same time, neutrophils produced ITA to negatively regulate NETs formation via inhibiting the expression of PAD4 and further Th17/IL-17 responses. Additionally, secreted ITA directly impeded the Th17 cell differentiation. ITA plays an important role in maintaining immune and inflammation balance in corticosteroid-resistant asthma. As an endogenous metabolite with low immunogenicity and toxicity, ITA presents substantial therapeutic potential for the management of patients suffering from corticosteroid-resistant asthma.

## Supplementary Material

Supplementary figures and tables.

## Data Availability

The data and information supporting the findings of this study are available in the main text and Supplementary Material.

## References

[1] Global Initiative for Asthma Global Strategy for Asthma Management and Prevention. 2024.

[2] Melén E, Zar HJ, Siroux V, Shaw D, Saglani S, Koppelman GH (2024). Asthma Inception: Epidemiologic Risk Factors and Natural History Across the Life Course. Am J Respir Crit Care Med.

[3] Huang K, Yang T, Xu J, Yang L, Zhao J, Zhang X (2019). Prevalence, risk factors, and management of asthma in China: a national cross-sectional study. Lancet.

[4] Guan WJ, Zheng XY, Chung KF, Zhong NS (2016). Impact of air pollution on the burden of chronic respiratory diseases in China: time for urgent action. Lancet.

[5] Guarnieri M, Balmes JR (2014). Outdoor air pollution and asthma. Lancet.

[6] Brandt EB, Kovacic MB, Lee GB, Gibson AM, Acciani TH, Le Cras TD (2013). Diesel exhaust particle induction of IL-17A contributes to severe asthma. J Allergy Clin Immunol.

[7] Bourdin A, Brusselle G, Couillard S, Fajt ML, Heaney LG, Israel E (2024). Phenotyping of Severe Asthma in the Era of Broad-Acting Anti-Asthma Biologics. J Allergy Clin Immunol Pract.

[8] O'Neill S, Sweeney J, Patterson CC, Menzies-Gow A, Niven R, Mansur AH (2015). The cost of treating severe refractory asthma in the UK: an economic analysis from the British Thoracic Society Difficult Asthma Registry. Thorax.

[9] Rider CF, Carlsten C (2019). Air pollution and resistance to inhaled glucocorticoids: Evidence, mechanisms and gaps to fill. Pharmacol Ther.

[10] Holgate ST, Polosa R (2006). The mechanisms, diagnosis, and management of severe asthma in adults. Lancet.

[11] Wenzel SE (2021). Severe Adult Asthmas: Integrating Clinical Features, Biology, and Therapeutics to Improve Outcomes. Am J Respir Crit Care Med.

[12] Zhang Q, Fu X, Wang C, Shen H, Zhu L, Shi G (2022). Severe eosinophilic asthma in Chinese C-BIOPRED asthma cohort. Clin Transl Med.

[13] Shaw DE, Sousa AR, Fowler SJ, Fleming LJ, Roberts G, Corfield J (2015). Clinical and inflammatory characteristics of the European U-BIOPRED adult severe asthma cohort. Eur Respir J.

[14] Tsai CH, Lai AC, Lin YC, Chi PY, Chen YC, Yang YH (2023). Neutrophil extracellular trap production and CCL4L2 expression influence corticosteroid response in asthma. Sci Transl Med.

[15] Ye D, Wang P, Chen LL, Guan KL, Xiong Y (2024). Itaconate in host inflammation and defense. Trends Endocrinol Metab.

[16] Shi X, Zhou H, Wei J, Mo W, Li Q, Lv X (2022). The signaling pathways and therapeutic potential of itaconate to alleviate inflammation and oxidative stress in inflammatory diseases. Redox Biol.

[17] Michelucci A, Cordes T, Ghelfi J, Pailot A, Reiling N, Goldmann O (2013). Immune-responsive gene 1 protein links metabolism to immunity by catalyzing itaconic acid production. Proc Natl Acad Sci U S A.

[18] Lampropoulou V, Sergushichev A, Bambouskova M, Nair S, Vincent EE, Loginicheva E (2016). Itaconate Links Inhibition of Succinate Dehydrogenase with Macrophage Metabolic Remodeling and Regulation of Inflammation. Cell Metab.

[19] Mills EL, Ryan DG, Prag HA, Dikovskaya D, Menon D, Zaslona Z (2018). Itaconate is an anti-inflammatory metabolite that activates Nrf2 via alkylation of KEAP1. Nature.

[20] Runtsch MC, Angiari S, Hooftman A, Wadhwa R, Zhang Y, Zheng Y (2022). Itaconate and itaconate derivatives target JAK1 to suppress alternative activation of macrophages. Cell Metab.

[21] Chen LL, Morcelle C, Cheng ZL, Chen X, Xu Y, Gao Y (2022). Itaconate inhibits TET DNA dioxygenases to dampen inflammatory responses. Nat Cell Biol.

[22] Mo Y, Ye L, Cai H, Zhu GP, Wang J, Zhu MC (2021). SERPINB10 contributes to asthma by inhibiting the apoptosis of allergenic Th2 cells. Respir Res.

[23] Xie Y, Abel PW, Casale TB, Tu Y (2022). T(H)17 cells and corticosteroid insensitivity in severe asthma. J Allergy Clin Immunol.

[24] Noval Rivas M, Chatila TA (2016). Regulatory T cells in allergic diseases. J Allergy Clin Immunol.

[25] Li P, Li M, Lindberg MR, Kennett MJ, Xiong N, Wang Y (2010). PAD4 is essential for antibacterial innate immunity mediated by neutrophil extracellular traps. J Exp Med.

[26] Ricciardolo FLM, Carriero V, Bullone M (2020). MicroRNAs as Biomarkers in Corticosteroid-Resistant/Neutrophilic Asthma: Still a Long Way to Go!. Am J Respir Crit Care Med.

[27] Zeng Y, Bai X, Zhu G, Zhu M, Peng W, Song J (2024). m(6)A-mediated HDAC9 upregulation promotes particulate matter-induced airway inflammation via epigenetic control of DUSP9-MAPK axis and acts as an inhaled nanotherapeutic target. J Hazard Mater.

[28] Brandt EB, Bolcas PE, Ruff BP, Khurana Hershey GK (2020). IL33 contributes to diesel pollution-mediated increase in experimental asthma severity. Allergy.

[29] Shin J, Kim J, Ham S, Choi S, Lee C, Lee J (2021). A unique population of neutrophils generated by air pollutant-induced lung damage exacerbates airway inflammation. The Journal of allergy and clinical immunology.

[30] Sun L, Fu J, Lin S, Sun J, Xia L, Lin C (2020). Particulate matter of 2.5 μm or less in diameter disturbs the balance of T17/regulatory T cells by targeting glutamate oxaloacetate transaminase 1 and hypoxia-inducible factor 1α in an asthma model. The Journal of allergy and clinical immunology.

[31] Zeng YR, Song JB, Wang D, Huang ZX, Zhang C, Sun YP (2023). The immunometabolite itaconate stimulates OXGR1 to promote mucociliary clearance during the pulmonary innate immune response. J Clin Invest.

[32] Sun KA, Li Y, Meliton AY, Woods PS, Kimmig LM, Cetin-Atalay R (2020). Endogenous itaconate is not required for particulate matter-induced NRF2 expression or inflammatory response. Elife.

[33] Tomlinson KL, Riquelme SA, Baskota SU, Drikic M, Monk IR, Stinear TP (2023). Staphylococcus aureus stimulates neutrophil itaconate production that suppresses the oxidative burst. Cell Rep.

[34] Rong K, Wang D, Pu X, Zhang C, Zhang P, Cao X (2025). Inflammatory macrophage-derived itaconate inhibits DNA demethylase TET2 to prevent excessive osteoclast activation in rheumatoid arthritis. Bone Res.

[35] Yin S, Tao Y, Li T, Li C, Cui Y, Zhang Y (2024). Itaconate facilitates viral infection via alkylating GDI2 and retaining Rab GTPase on the membrane. Signal Transduct Target Ther.

[36] Shan M, Zhang S, Luo Z, Deng S, Ran L, Zhou Q (2025). Itaconate promotes inflammatory responses in tissue-resident alveolar macrophages and exacerbates acute lung injury. Cell Metab.

[37] Sørensen OE, Borregaard N (2016). Neutrophil extracellular traps - the dark side of neutrophils. J Clin Invest.

[38] Papayannopoulos V (2018). Neutrophil extracellular traps in immunity and disease. Nat Rev Immunol.

[39] Krishnamoorthy N, Douda DN, Brüggemann TR, Ricklefs I, Duvall MG, Abdulnour RE (2018). Neutrophil cytoplasts induce T(H)17 differentiation and skew inflammation toward neutrophilia in severe asthma. Sci Immunol.

[40] Lachowicz-Scroggins ME, Dunican EM, Charbit AR, Raymond W, Looney MR, Peters MC (2019). Extracellular DNA, Neutrophil Extracellular Traps, and Inflammasome Activation in Severe Asthma. Am J Respir Crit Care Med.

[41] Liu J, Tao P, Su B, Zheng L, Lin Y, Zou X (2025). Interleukin-33 modulates NET formation via an autophagy-dependent manner to promote neutrophilic inflammation in cigarette smoke-exposure asthma. J Hazard Mater.

[42] Wilson AS, Randall KL, Pettitt JA, Ellyard JI, Blumenthal A, Enders A (2022). Neutrophil extracellular traps and their histones promote Th17 cell differentiation directly via TLR2. Nat Commun.

[43] Kim TS, Silva LM, Theofilou VI, Greenwell-Wild T, Li L, Williams DW (2023). Neutrophil extracellular traps and extracellular histones potentiate IL-17 inflammation in periodontitis. J Exp Med.

[44] Wang H, Kim SJ, Lei Y, Wang S, Wang H, Huang H (2024). Neutrophil extracellular traps in homeostasis and disease. Signal Transduct Target Ther.

[45] Liu H, Jing G, Wu S, Yuan M, Dong Y, Chen X Acod1 Promotes PAD4 Ubiquitination via UBR5 Alkylation to Modulate NETosis and Exert Protective Effects in Sepsis. Adv Sci (Weinh). 2025: e11652.

[46] Aso K, Kono M, Kanda M, Kudo Y, Sakiyama K, Hisada R (2023). Itaconate ameliorates autoimmunity by modulating T cell imbalance via metabolic and epigenetic reprogramming. Nat Commun.

