# Supplementary material for: Itaconate Modulates Neutrophil Homeostasis to Ameliorate Airway Inflammation in Diesel Exhaust Particles-exacerbated Asthma via Inhibiting NETs Formation
Source: Int J Biol Sci. 2026 Feb 11;22(5):2603–21. doi: 10.7150/ijbs.124927 (PMC12965240; doi:10.7150/ijbs.124927)
Supplement: Supplementary file 1 — Supplementary figures and tables. [file ijbsv22p2603s1.pdf]

Supplementary Materials for

Itaconate modulates neutrophil homeostasis to ameliorate airway inflammation in corticosteroid-resistant asthma via inhibiting NETs formation

Guiping Zhu *et al.*

*Corresponding author. Email: jian.wang5@zs-hospital.sh.cn

Supplementary Text

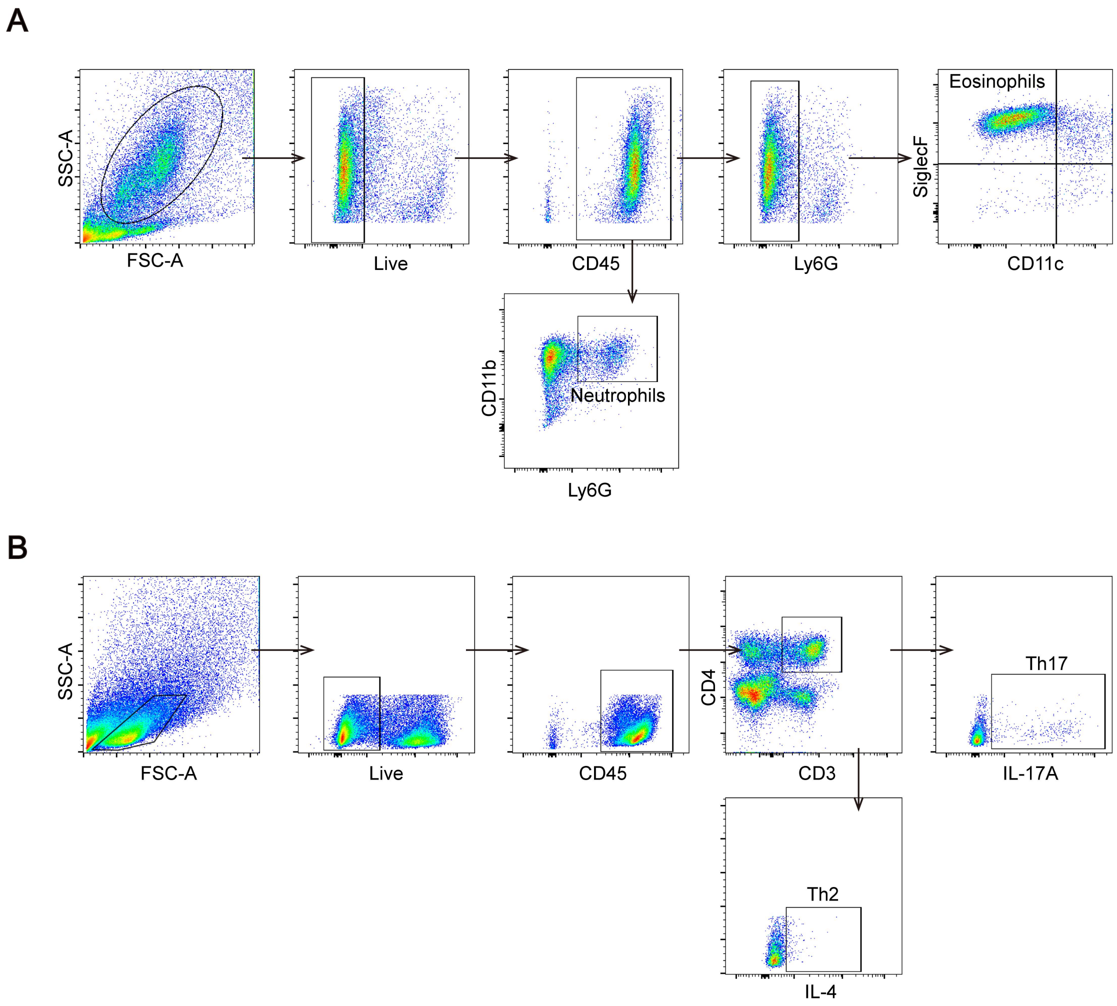

Fig. S1. Flow cytometry gating strategy of inflammatory cells in BALF and Th cells in the lung tissues. (A) Flow cytometry gating strategy for inflammatory cells in BALF. Live cells can be separated in a $CD45^{+}$ and a $CD45^{-}$ cell population. Gating eosinophils ($Ly6G^{-}CD11c^{-}SiglecF^{+}$) and neutrophils ($Ly6G^{+}CD11b^{+}$) within the $CD45^{+}$ cell population. (B) Flow cytometry gating strategy for Th2 and Th17 cells in the lung tissues. Live cells can be separated in a $CD45^{+}$ and a $CD45^{-}$ cell population. Gating $CD3^{+}CD4^{+}$ within the $CD45^{+}$ cell population. Then Th2 cells were gated as IL-4^{+}, and Th17 cells were gated as IL-$17A^{+}$.

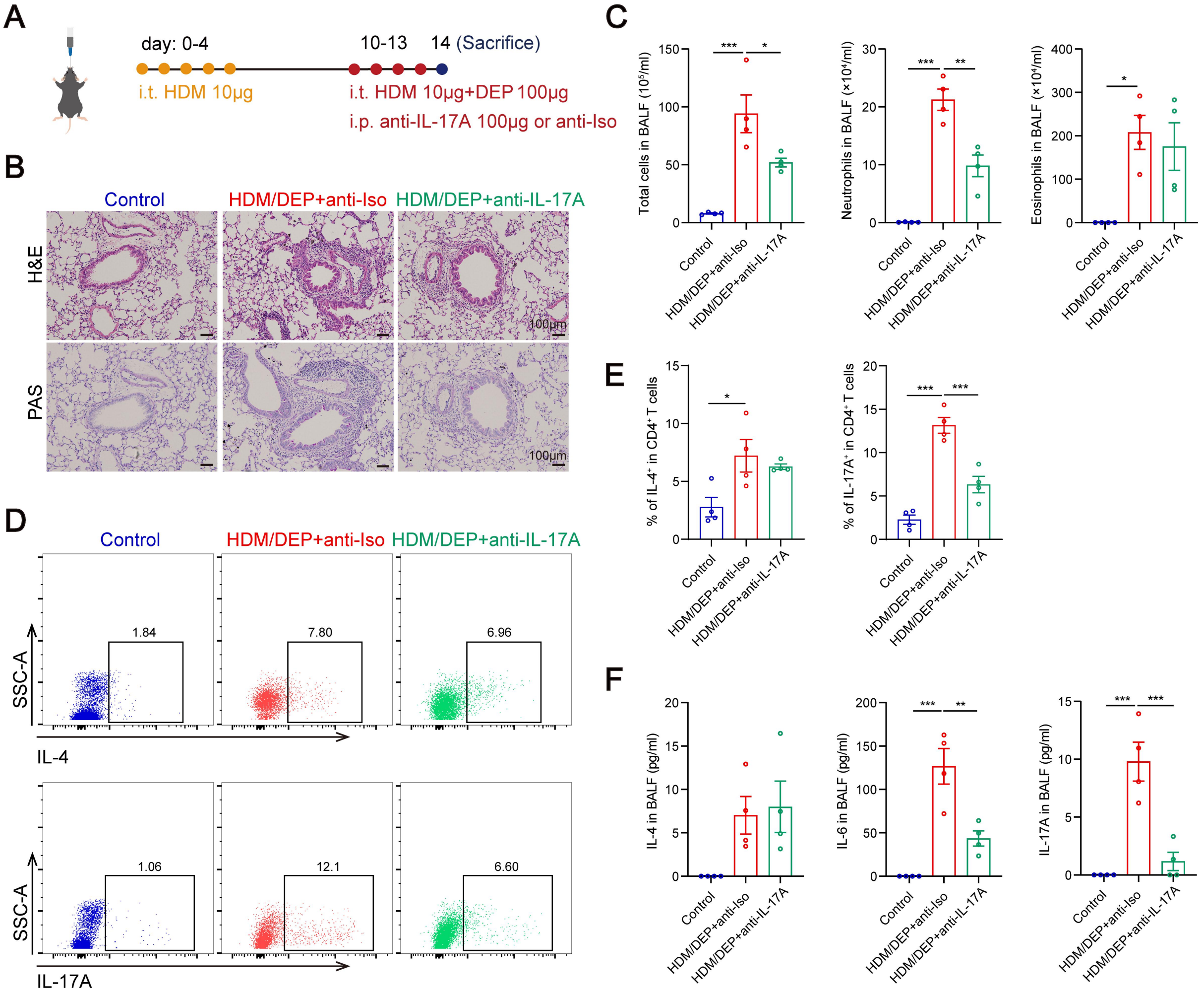

Fig. S2. Neutralization of IL-17A inhibits airway inflammation in HDM/DEP-induced corticosteroid-resistant asthma. (A) For neutralization of IL-17A, anti-IL-17A antibody was administered 1 hour before HDM and DEP challenge. (B) Representative H&E and PAS staining pictures of lung tissues (n = 4 mice per group). Scale bar, 100 μm. (C) Total cell, neutrophil, and eosinophil counts in BALF measured by flow cytometry (n = 4 mice per group). (D and E) The percentage of IL-4^{+} (Th2) and IL-17A^{+} (Th17) cells in CD4^{+} T cells (gated on Live, CD45^{+}CD3^{+}CD4^{+}) measured using flow cytometry (n = 4 mice per group). (F) Levels of inflammatory cytokines in BALF measured by using a cytometric bead array (n = 4 mice per group). Data are presented as means ± SEM. $^{*}P < 0.05$, $^{**}P < 0.01$, $^{***}P < 0.001$. i.t., intratracheally; i.p., intraperitoneally; HDM, house dust mite; DEP, diesel exhaust particles; BALF, bronchoalveolar lavage fluid.

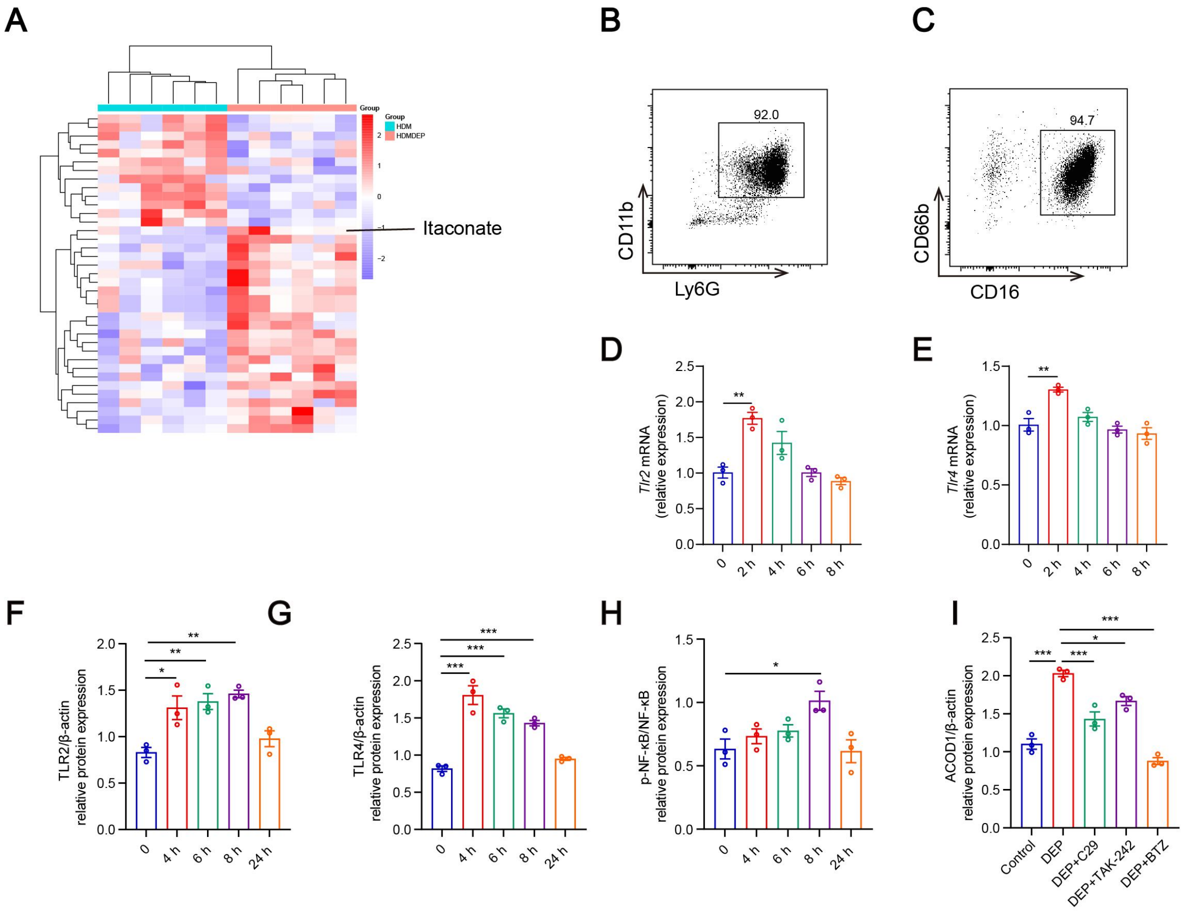

Fig. S3. (A) Heatmap of differentially accumulated metabolites between HDM and HDM/DEP groups. (B) Isolation of neutrophils from mouse bone marrow, with cell purity assessed by flow cytometry. Neutrophils were identified as CD45$^+$Ly6G$^+$CD11b$^+$. (C) Isolation of neutrophils from human peripheral blood, with cell purity assessed by flow cytometry. Neutrophils were identified as CD45$^+$CD16$^+$CD66b$^+$. (D) Real-time qPCR analysis of *Tlr2* in BMDNs stimulated with DEP at different time points. (E) Real-time qPCR analysis of *Tlr4* in BMDNs stimulated with DEP at different time points. (F) Quantitative analysis of TLR2 expression in BMDNs stimulated with DEP, normalized to β-actin. (G) Quantitative analysis of TLR4 expression in BMDNs stimulated with DEP, normalized to β-actin. (H) Quantitative analysis of p-NF-κB expression in BMDNs stimulated with DEP, normalized to NF-κB. (I) Quantitative analysis of ACOD1 expression in BMDNs stimulated with DEP and treated with inhibitors of TLR2, TLR4, and NF-κB pathway, normalized to β-actin. Data are presented as means ± SEM. $*P < 0.05$, $**P < 0.01$, $***P < 0.001$. HDM, house dust mite; DEP, diesel exhaust particles; BMDNs, bone

marrow-derived neutrophils; Tlr, toll-like receptor; ACOD1, aconitate decarboxylase 1; BTZ, Bortezomib.

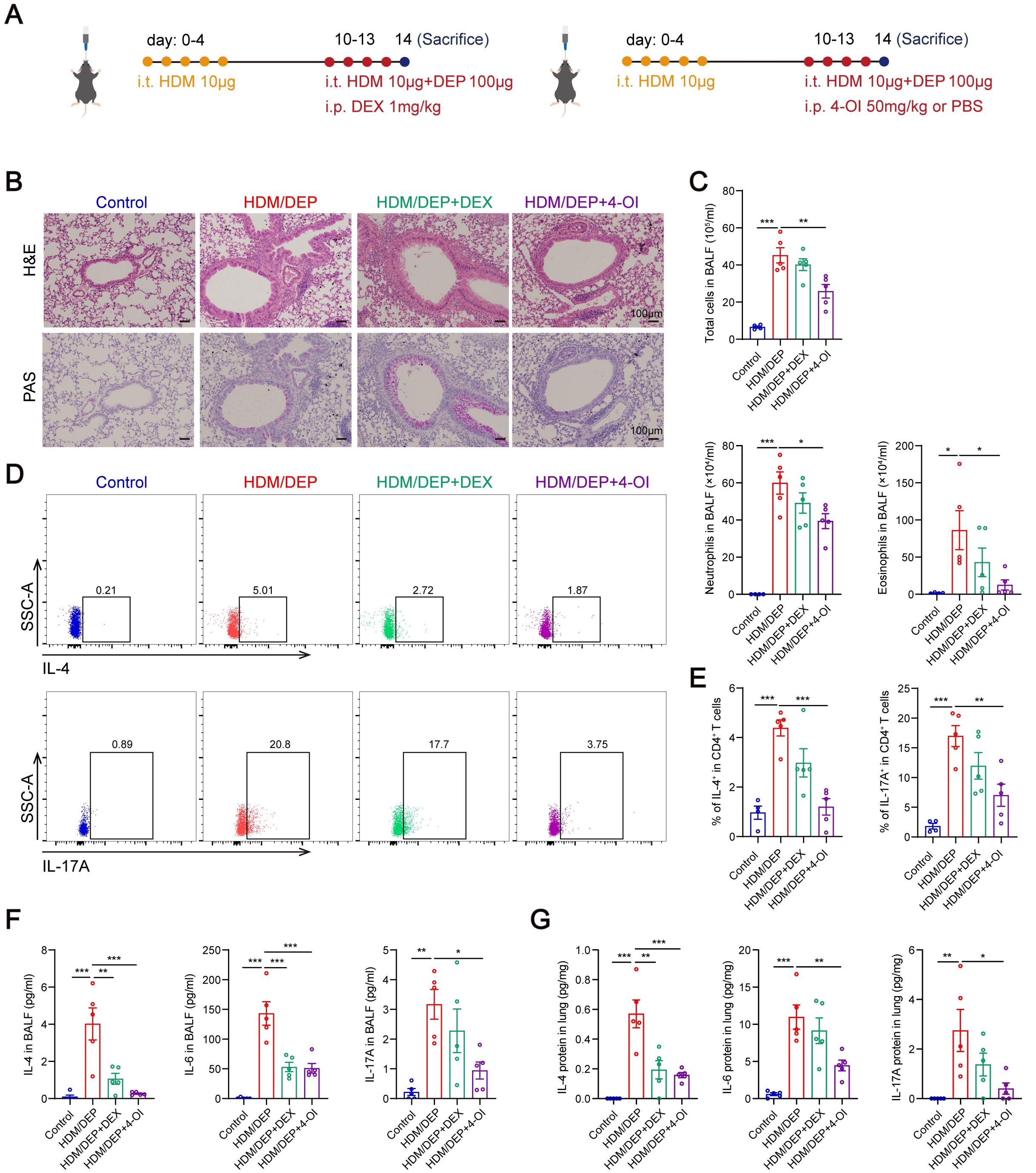

Fig. S4. 4-OI suppresses airway inflammation in HDM/DEP-induced corticosteroid-resistant asthma. (A) HDM/DEP-induced asthmatic mice were established. 4-OI was administered 1 hour before HDM and DEP challenge. (B) Representative H&E and PAS staining pictures of lung tissues (n = 5 mice per group). Scale bar, 100 μm. (C) Total cell, neutrophil, and

eosinophil counts in BALF measured by flow cytometry (n = 4-5 mice per group). (D and E) The percentage of IL-4^+ (Th2) and IL-$17A^+$ (Th17) cells in $CD4^+$ T cells (gated on Live, $CD45^+CD3^+CD4^+$) measured using flow cytometry (n = 4-5 mice per group). (F) Levels of inflammatory cytokines in BALF measured by using a cytometric bead array (n = 5 mice per group). (G) Levels of inflammatory cytokines in lung homogenates measured by using a cytometric bead array (n = 5 mice per group). Data are presented as means ± SEM. $^{*}P < 0.05$, $^{**}P < 0.01$, $^{***}P < 0.001$. i.t., intratracheally; i.p., intraperitoneally; HDM, house dust mite; DEP, diesel exhaust particles; BALF, bronchoalveolar lavage fluid. DEX, dexamethasone; 4-OI, 4-octyl itaconate.

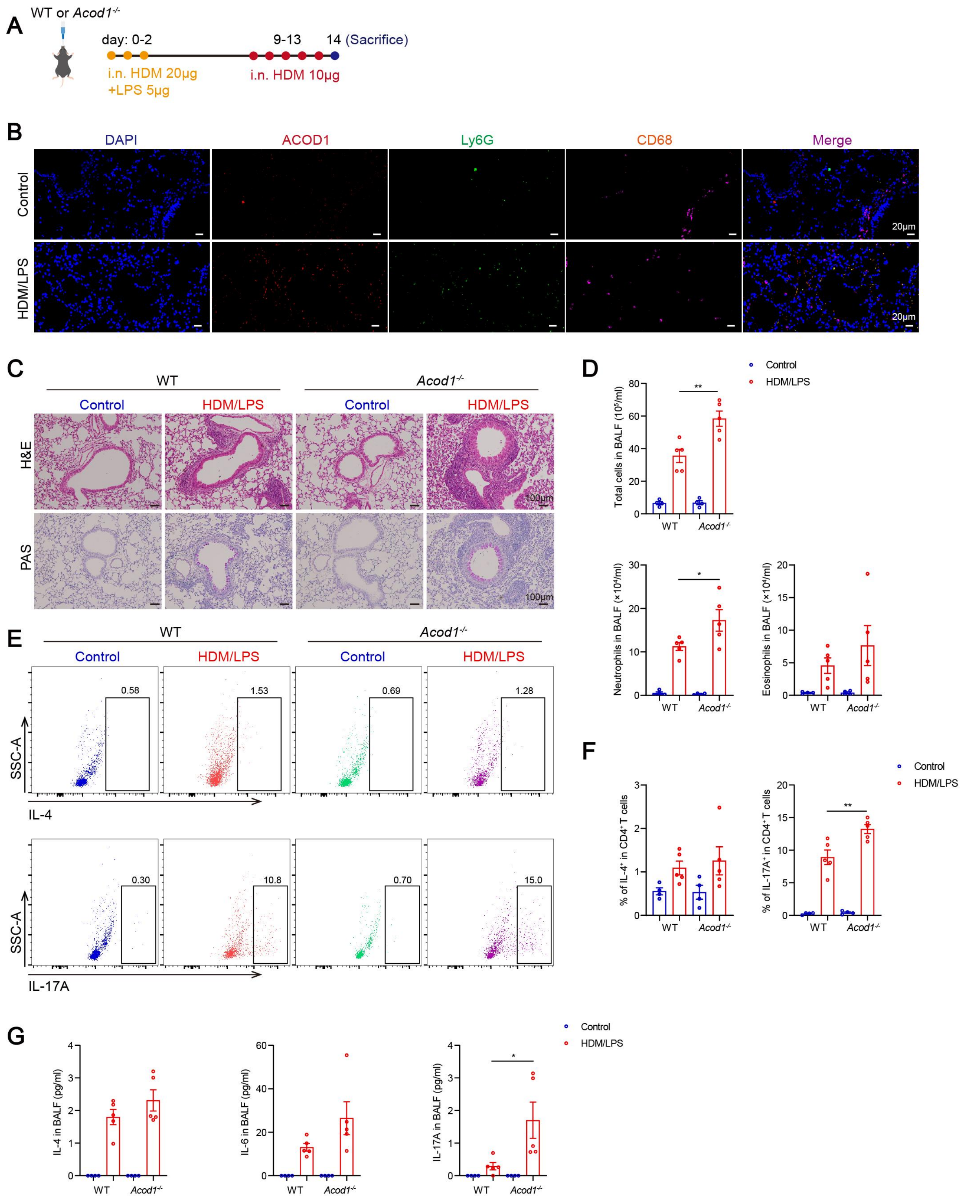

Fig. S5. ***Acod1*** **knockout exacerbates airway inflammation in HDM/LPS-induced corticosteroid-resistant asthma.** (A) WT or *Acod1*$^{-/-}$ mice were sensitized with HDM and LPS,

followed by challenge with HDM. (B) Representative immunofluorescence staining of ACOD1 in the lung tissues of LPS-induced asthmatic mice. Ly6G was used to mark neutrophils, and CD68 to mark macrophages. ACOD1 was co-localized with neutrophils. Scale bar, 20 μm. (C) Representative H&E and PAS staining pictures of lung tissues (n = 4-5 mice per group). Scale bar, 100 μm. (D) Total cell, neutrophil, and eosinophil counts in BALF measured by flow cytometry (n = 4-5 mice per group). (E and F) The percentage of IL-4$^+$ (Th2) and IL-17A$^+$ (Th17) cells in CD4$^+$ T cells (gated on Live, CD45$^+$CD3$^+$CD4$^+$) measured using flow cytometry (n = 4-5 mice per group). (G) Levels of inflammatory cytokines in BALF measured by using a cytometric bead array (n = 4-5 mice per group). Data are presented as means ± SEM. $^*P < 0.05$, $^{**}P < 0.01$, $^{***}P < 0.001$. i.n. intranasally; HDM, house dust mite; LPS, lipopolysaccharide; ACOD1, aconitate decarboxylase 1; BALF, bronchoalveolar lavage fluid.

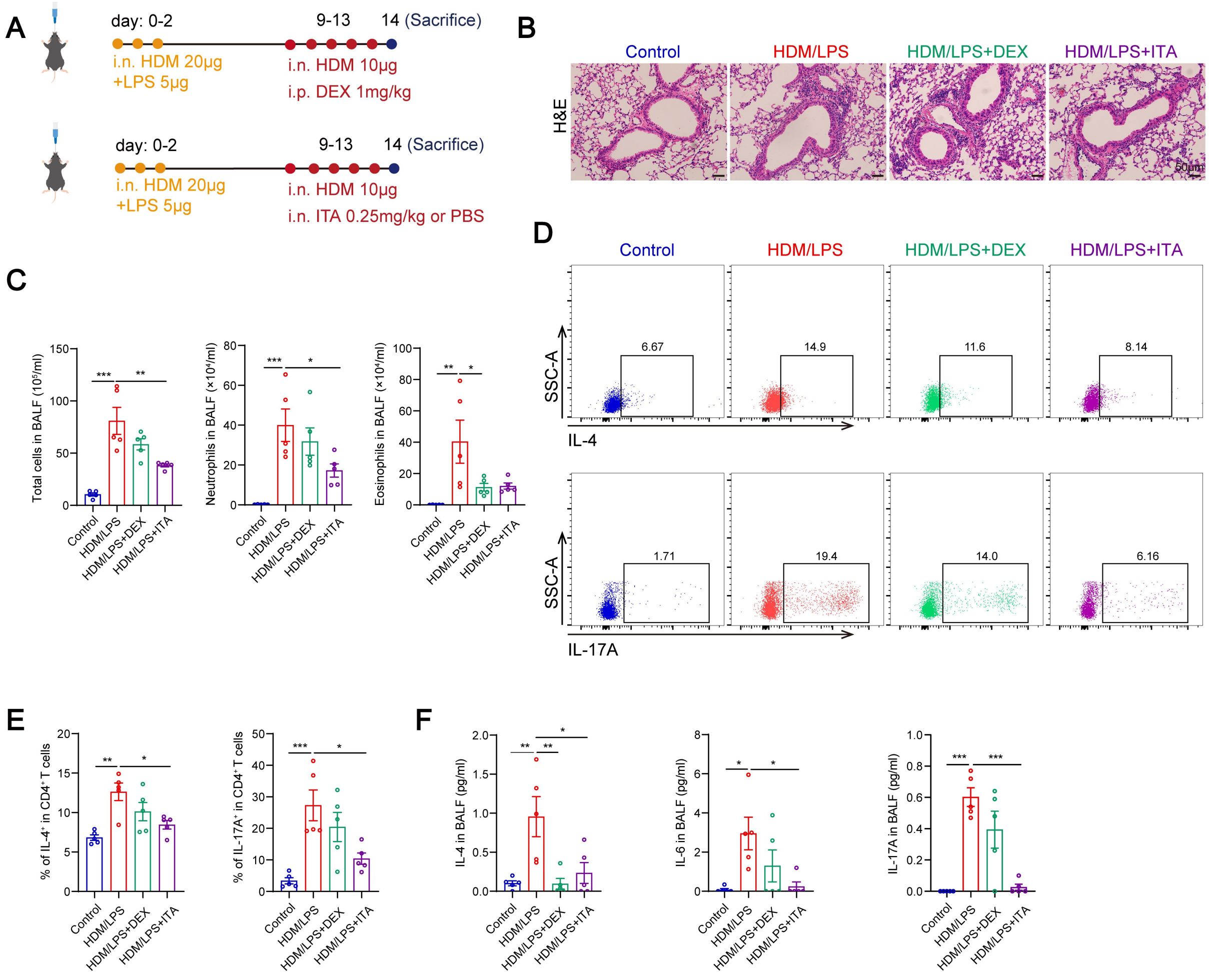

Fig. S6. ITA inhibits airway inflammation in HDM/LPS-induced corticosteroid-resistant asthma. (A) HDM/LPS-induced corticosteroid-resistant asthma was established. DEX or ITA was administered 1 hour before HDM challenge. (B) Representative H&E staining pictures of lung tissues (n = 5 mice per group). Scale bar, 50 μm. (C) Total cell, neutrophil, and eosinophil counts in BALF measured by flow cytometry (n = 5 mice per group). (D and E) The percentage of IL-4$^+$ (Th2) and IL-17A$^+$ (Th17) cells in CD4$^+$ T cells (gated on Live, CD45$^+$CD3$^+$CD4$^+$) measured using flow cytometry (n = 5 mice per group). (F) Levels of inflammatory cytokines in BALF measured by using a cytometric bead array (n = 5 mice per group). Data are presented as means ± SEM. $^*P < 0.05$, $^{**}P < 0.01$, $^{***}P < 0.001$. i.n. intranasally; i.p., intraperitoneally; HDM, house dust mite; LPS, lipopolysaccharide; DEX, dexamethasone; ITA, itaconate; BALF, bronchoalveolar lavage fluid.

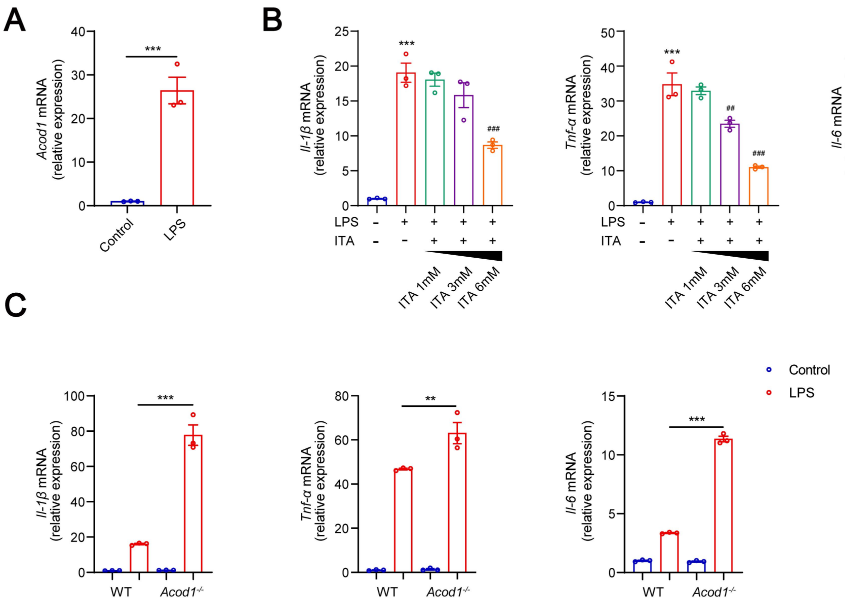

Fig. S7. ITA suppresses inflammatory responses in neutrophils stimulated by LPS. (A) Real-time qPCR analysis of *Acod1* in BMDNs stimulated with LPS. (B) Real-time qPCR analysis of inflammatory cytokines (*Il-1β*, *Tnf-α*, and *Il-6*) in BMDNs stimulated with LPS and treated with ITA. $^{*}P < 0.05$, $^{**}P < 0.01$, $^{***}P < 0.001$, compared with the control group. $^{\#}P < 0.05$, $^{\#\#}P < 0.01$, $^{\#\#\#}P < 0.001$, compared with the LPS group. (C) Real-time qPCR analysis of inflammatory cytokines (*Il-1β*, *Tnf-α*, and *Il-6*) in LPS-stimulated BMDNs from WT or *Acod1*$^{-/-}$ mice. Data are presented as means ± SEM. $^{*}P < 0.05$, $^{**}P < 0.01$, $^{***}P < 0.001$. LPS, lipopolysaccharide; ITA, itaconate; Acod1, aconitate decarboxylase 1; BMDNs, bone marrow-derived neutrophils.

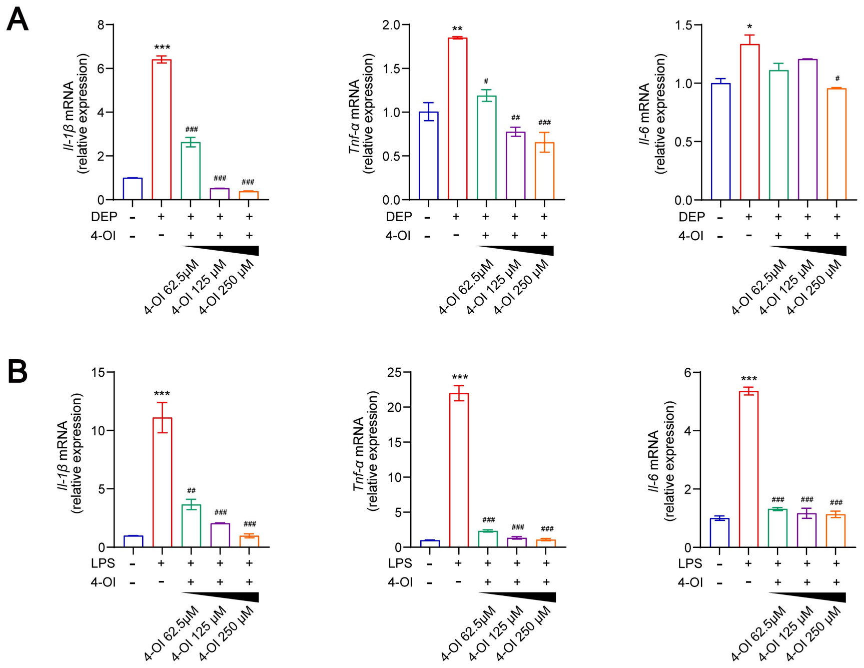

Fig. S8. 4-OI suppresses inflammatory responses in neutrophils stimulated by DEP or LPS. (A) Real-time qPCR analysis of inflammatory cytokines (*Il-1β*, *Tnf-α*, and *Il-6*) in BMDNs stimulated with DEP and treated with 4-OI. (B) Real-time qPCR analysis of inflammatory cytokines (*Il-1β*, *Tnf-α*, and *Il-6*) in BMDNs stimulated with LPS and treated with 4-OI. Data are presented as means ± SEM. $^{*}P < 0.05$, $^{**}P < 0.01$, $^{***}P < 0.001$, compared with the control group. $^{\#}P < 0.05$, $^{\#\#}P < 0.01$, $^{\#\#\#}P < 0.001$, compared with the DEP or LPS group. BMDNs, bone marrow-derived neutrophils; LPS, lipopolysaccharide; DEP, diesel exhaust particles; 4-OI, 4-octyl itaconate.

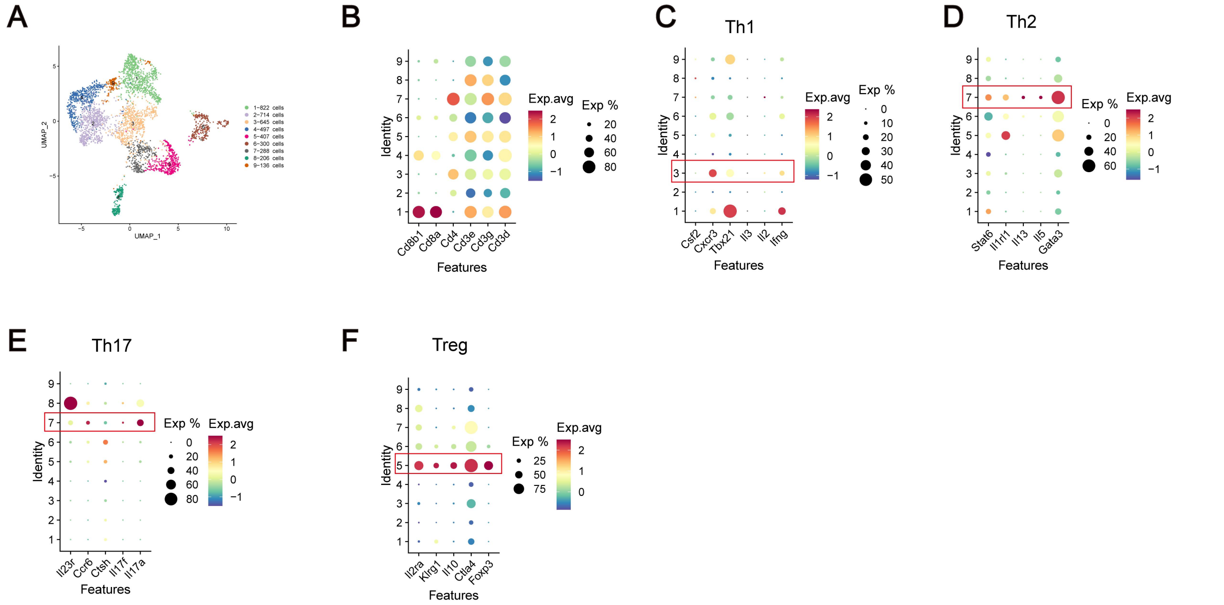

Fig. S9. Identification of T cell clusters based on characteristic genes. (A) Uniform Manifold Approximation and Projection (UMAP) plot showing clusters of T cells in the lung tissues. (B) Dotplot of marker genes in T cells. (C-F) Dotplot of representative marker genes in Th1, Th2, Th17, and Treg cells

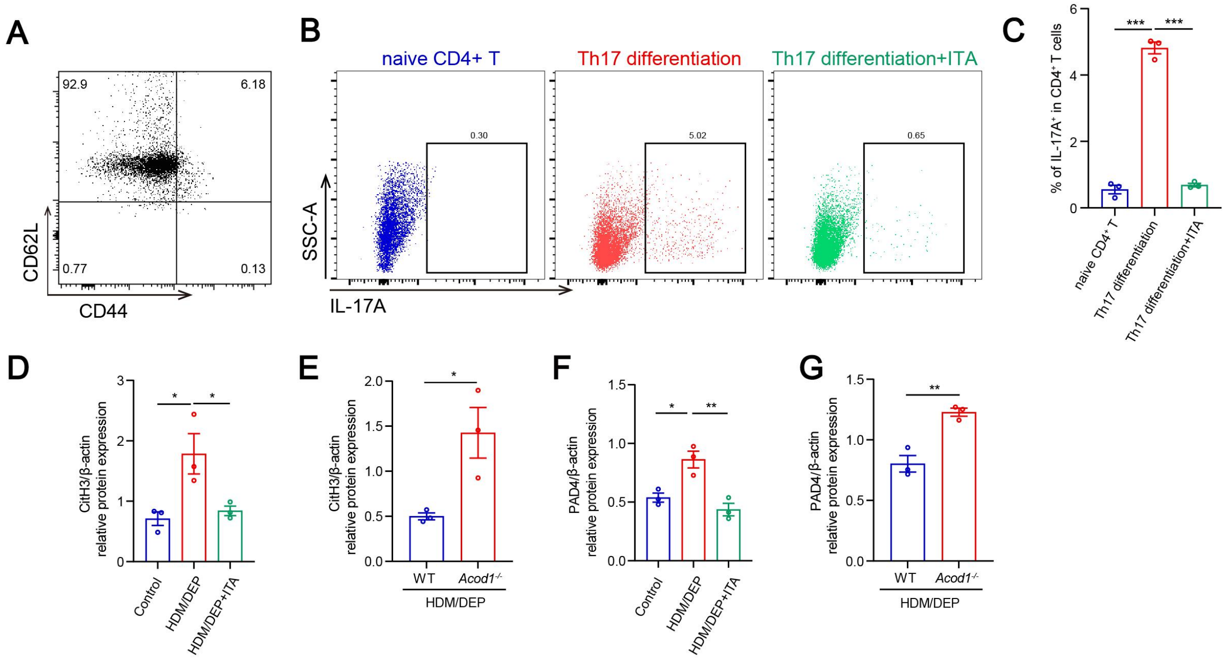

Fig. S10. ITA directly inhibits Th17 cell differentiation. (A) Isolation of mouse naive CD4$^+$ T cells from mouse spleen, with cell purity assessed by flow cytometry. Naive CD4$^+$ T cells were identified as CD4$^+$CD44$^-$CD62L$^+$. (B-C) Naive CD4$^+$ T cells were cultured under Th17 differentiation condition and treated with ITA. The percentage of IL-17A$^+$ (Th17) cells in CD4$^+$ T cells measured using flow cytometry. (D) Quantitative analysis of CitH3 expression in the lung tissues of HDM/DEP-induced asthma with or without treatment of ITA, normalized to β-actin. (E) Quantitative analysis of CitH3 expression in the lung tissues of HDM/DEP-induced asthma in WT or *Acod1*$^{-/-}$ mice, normalized to β-actin. (F) Quantitative analysis of PAD4 expression in the lung tissues of HDM/DEP-induced asthma with or without treatment of ITA, normalized to β-actin. (G) Quantitative analysis of PAD4 expression in the lung tissues of HDM/DEP-induced asthma in WT or *Acod1*$^{-/-}$ mice, normalized to β-actin. Data are presented as means ± SEM. $^*P < 0.05$, $^{**}P < 0.01$, $^{***}P < 0.001$. HDM, house dust mite; DEP, diesel exhaust particles; ACOD1, aconitate decarboxylase 1; ITA, itaconate; CitH3, citrulline histone H3; PAD4, peptidylarginine deiminase 4.

Table S1. primer sequences used in this study.

Gene		
Mouse *Acod1*	Forward	AATGAAACCTTGGGTCTTATGCC
	Reverse	TGCCCATGACTTATCCAGACAG
Mouse *Gapdh*	Forward	GGGTGTGAACCACGAGAAAT
	Reverse	CCTTCCACAATGCCAAAGTT
Mouse *Il-6*	Forward	TAGTCCTTCCTACCCCAATTTCC
	Reverse	TTGGTCCTTAGCCACTCCTTC
Mouse *Tlr4*	Forward	AGGACTATGTGATGTGACCATTGATG
	Reverse	GATACACCTGCCAGAGACATTGC
Mouse *Tlr2*	Forward	TGGTGTCTGGAGTCTGCTGTG
	Reverse	GCTTTCTTGGGCTTCCTCTTGG
Mouse *Il-1β*	Forward	GCAACTGTTCCTGAACTCAACT
	Reverse	ATCTTTTGGGGTCCGTCAACT
Mouse *Tnf-α*	Forward	CCCTCACACTCAGATCATCTTCT
	Reverse	GCTACGACGTGGGCTACAG
Human *ACOD1*	Forward	GGTCTCCAAGGAAACAAGCAGGTC
	Reverse	CCAGCAGCCAACTGTAGGAAGC
Human *GAPDH*	Forward	GGAGCGAGATCCCTCCAAAAT
	Reverse	GGCTGTTGTCATACTTCTCATGG

Table S2. Baseline characteristics of healthy controls and patients with mild-to-moderate or severe asthma.

	Healthy controls (n=18)	Mild-to-moderate asthma (n=31)	Severe asthma (n=30)
Age, year	38.22 ±7.62	38.39 ±10.22	42.47 ±14.99
Sex, No. (%)			
Female	5 (27.78%)	21 (67.74)	16 (53.33%)
Male	13 (72.22%)	10 (32.26%)	14 (46.67%)
Blood neutrophil (%) (n=74)	59.4 ±5.221	57.81±8.774	57.72 ±9.248
Blood neutrophil (/µL) (n=74)	3950 (3175, 4850)	3900 (3300,4600)	4700 (3200,6450)
Blood eosinophil (%) (n=74)	2.35 (1.2, 3.325)	3.3 (1.4, 5.3)	5.7 (2.15, 8.9)
Blood eosinophil (/µL) (n=74)	145 (87.5,212.5)	200 (90, 430)	440 (155, 725)
